# Simulating Genetic Mixing in Strongly Structured Populations of the Threatened Southern Brown Bandicoot (*Isoodon obesulus*)

**DOI:** 10.1111/eva.70050

**Published:** 2024-12-05

**Authors:** John G. Black, Steven J. B. Cooper, Thomas L. Schmidt, Andrew R. Weeks

**Affiliations:** ^1^ School of Biosciences The University of Melbourne Melbourne Victoria Australia; ^2^ School of Biological Sciences and the Environment Institute The University of Adelaide Adelaide South Australia Australia; ^3^ South Australian Museum Adelaide South Australia Australia; ^4^ Cesar Australia Brunswick Victoria Australia

**Keywords:** conservation genomics, genetic augmentation, isolation, marsupial, population genetics, population mixing

## Abstract

Genetic mixing aims to increase the genetic diversity of small or isolated populations, by mitigating genetic drift and inbreeding depression, either by maximally increasing genetic diversity, or minimising the prevalence of recessive, deleterious alleles. However, few studies investigate this beyond a single generation of mixing. Here, we model genetic mixing using captive, low‐diversity recipient population of the threatened Southern brown bandicoot (*Isoodon obesulus*) over 50 generations and compare wild populations across south‐eastern Australia as candidate source populations. We first assess genetic differentiation between 12 populations, including the first genomic assessment of three mainland Australian and three Tasmanian populations. We assess genetic diversity in the 12 populations using an individualised autosomal heterozygosity pipeline, using these results to identify a candidate recipient population for genetic mixing simulations. We found that populations fell into four major groups of genetic similarity: Adelaide Hills, western Victoria, eastern Victoria, and Tasmania, but populations within these groups were also distinct, and additional substructure was observed in some populations. Our autosomal heterozygosity pipeline indicated significant variability in mean heterozygosity between populations, identifying one extremely genetically degraded population on Inner Sister Island, Tasmania. Genetic mixing simulations of a low heterozygosity captive population in Victoria suggested the greatest increase in heterozygosity would be reached by using highly differentiated populations as mixing sources. However, when removing populations that may represent taxonomically discrete lineages, neither metrics of differentiation nor heterozygosity was strongly correlated with modelled heterozygosity increase, indicating the value of simulation‐based approaches when selecting source populations for population mixing.

## 1 Introduction

In the face of anthropogenic habitat fragmentation, translocations are becoming an increasingly common tool to combat the resulting losses of genetic diversity (Resende et al. 2020). While the effects of fragmentation on biodiversity are complex, a common outcome of population isolation resulting from fragmentation is lower effective and census population size in isolated sub‐regions (Fahrig 2017; Fletcher et al. 2018; Haddad et al. 2015; Püttker et al. 2020; Rybicki, Abrego, and Ovaskainen 2020). Furthermore, as anthropogenic fragmentation decreases the proportion of habitat with sufficient complexity to provide cover for prey species, they become more vulnerable to predation in fragmented habitats (Rees and Paull 2000). Predators have also been observed utilising cleared corridors, such as roads (Frey and Conover 2006), which further modifies prey species behaviour (Mata, Herranz, and Malo 2020).

As population size decreases, habitat fragmentation then catalyses the loss of genetic variation due to genetic drift, leading to maladaptation and an increased likelihood of inbreeding, with insufficient individuals remaining for mutation to shore up genetic diversity (Lynch et al. 2016). Inbreeding then causes recessive, deleterious alleles to become homozygous and phenotypically expressed in offspring, and so a subsequent feedback loop of increased population decline followed by even more extreme inbreeding and genetic drift can occur (Fagan and Holmes 2006; Gilpin and Soulé 1986). Increased homozygosity has been found to exhibit fitness consequences including lower longevity, lower breeding success, reduced ability to adapt to environmental change, and lower resilience to parasites and pathogens (Hoffmann, Sgrò, and Kristensen 2017; Stewart et al. 2017; Weeks, Stoklosa, and Hoffmann 2016). Genetic drift can also fix recessive, deleterious alleles, resulting in increased genetic load and maladaptation (Lynch et al. 2016; Mathur, Tomeček, et al. 2023; Stewart et al. 2017). While deleterious alleles can be hard to detect, neutral genetic variation is generally associated with functional diversity (Mathur, Mason, et al. 2023; Willi et al. 2022), and as reductions in this variation often occur before population crashes (Spielman, Brook, and Frankham 2004), it can represent an early warning sign that a population is in need of genetic management.

To reverse the effects of inbreeding and genetic drift within a population that is not experiencing natural gene flow, new genetic variation is required, which can be achieved by growing the populations' size to increase the volume of novel alleles produced by mutation (Lynch et al. 2016), moving new individuals from the same species into the depauperate population in a process known as genetic mixing or augmentation (Hoffmann, Miller, and Weeks 2021), or restoring gene flow through habitat reconnection (cite). For mutation to stabilise genetic drift and combat maladapted genotypes, populations must be sufficiently large (Frankham, Bradshaw, and Brook 2014), which itself requires habitat that may not be available, especially if a population is at an intrinsically limited location such as a predator‐proofed reserve or island. For populations that cannot expand, the only way to introduce novel genetic diversity is through the migration of animals into that population, such as restoring wildlife corridors to reconnect fragmented habitats or facilitated translocations of animals (Hoffmann et al. 2015), and subsequent interbreeding. If habitat can be reconnected, the populations in combination may be a self‐sustaining size and therefore prevent future loss of genetic diversity, but if facilitated translocation into a habitat‐restricted location is required, this may have to be repeated after several generations as genetic diversity will continue to slowly decline (Hedrick and Fredrickson 2010) and strong demographic effects can reduce the variation retained in later generations (Hedrick et al. 2019). The alternative of inaction, however, will likely lead to population extinction, which will further degrade the remaining genetic diversity and evolutionary potential of the species.

Genetic mixing refers to a variety of strategies which aim to stop population extinction, increase individual and population fitness, and facilitate adaptation, by mixing individuals from two or more populations (Hoffmann, Miller, and Weeks 2021). It is still debated whether the selection of the source population should simply be one with maximal overall genetic diversity (Ralls et al. 2020) or designed to minimise import of recessive, deleterious alleles as much as possible (Kyriazis, Wayne, and Lohmueller 2021). The former is considerably easier than the latter to predict and model, especially considering recessive variation may be masked by heterozygosity until populations are significantly contracted. Furthermore, population selection must also balance the potential of outbreeding depression to inhibit the success of genetic mixing (Byrne and Silla 2020; Montecinos et al. 2021), although the import of novel genetic variation to increase genetic diversity is now well supported by empirical evidence (Frankham 2015; Hoffmann, Miller, and Weeks 2021; Weeks et al. 2017; Whiteley et al. 2015).

Here, we assess population genetic structure and genomic diversity in a threatened marsupial, the southern brown bandicoot (*Isoodon obesulus*, Shaw, 1797), using genome‐wide SNP data from 171 individuals sampled from across its distribution in south‐eastern Australia. Using these data, we then simulate population mixing events to recommend which populations may be suitable for genetic mixing to restore diversity in a depauperate population. *Isoodon obesulus* represents a strong candidate for population mixing as remaining populations are fragmented, with variable levels of genetic differentiation and diversity, with smaller populations and fenced captive sites either known or inferred to have low diversity (Robinson et al. 2021). A strong signal of population structure has repeatedly been observed across the Murray River (Cooper et al. 2018; Thavornkanlapachai et al. 2021), and weaker population structure (and substructure) has been found between most populations (Li et al. 2016, 2014; Robinson et al. 2021), highlighting the fragmentation sensitivity of this species (Ramalho et al. 2018). Furthermore, several *I. obesulus* populations have declined significantly since 2000, particularly those to the east of Melbourne, Victoria, due to rapid urban and agricultural land conversion, and in more arid regions due to drought in combination with other anthropogenic stressors (Bachmann and Fullagar 2017; Bryant et al. 2018; Coates, Nicholls, and Willig 2008; Ralph 2021; Robinson et al. 2021). As a result, *I. obesulus* is also at “high” risk of genetic consequences, due to its sensitivity to habitat loss and fragmentation (Kriesner et al. 2020), making it a strong candidate for population mixing.

There has been taxonomic debate within the *Isoodon* genus, leading to the recent reclassification of two *I. obesulus* subspecies to species: *I. fusciventer* at south‐western West Australia (Cooper et al. 2018; Westerman et al. 2012) and *I. peninsulae* at Cape York (Cooper et al. 2018; Westerman et al. 2012; Zenger, Eldridge, and Johnston 2005). Proposals have been made that Adelaide Hills and nearby populations of *I. obesulus* should also be elevated to the distinction of subspecies or species (Cooper et al. 2018), which would significantly reduce the range and total size of *I. obesulus*. Tasmanian populations are currently designated as *I. o. affinis*; however, the only evidence for this is 2.3% mtDNA sequence divergence and paraphyly of NADH dehydrogenase 2 sequence with Victorian *I. obesulus* (Cooper et al. 2018). Although Cooper et al. (2018) support subspecies designation, they acknowledge that further investigations, including nuclear DNA, are required to confirm this.

This study identifies population structure and differentiation between several *I. obesulus* populations, including the first genomic assessment of three Tasmanian populations and two mainland Australian populations. We then assess population genetic diversity with an individualised, reference‐free autosomal heterozygosity pipeline designed for *de novo*‐aligned RADseq data but also applicable to reference‐aligned data. Finally, using these data, we select a candidate population for genetic mixing, and model a single mixing event and the resulting change in genetic diversity over 50 generations (~100 years), using all other populations as candidate sources, on the basis of using maximally increased genetic diversity to obviate the effects of small population size and fragmentation.

## 2 Methods

More details on the target species’ biology and ecology are available in Appendix S1.

### 2.1 Genetic Samples

**FIGURE 1:**
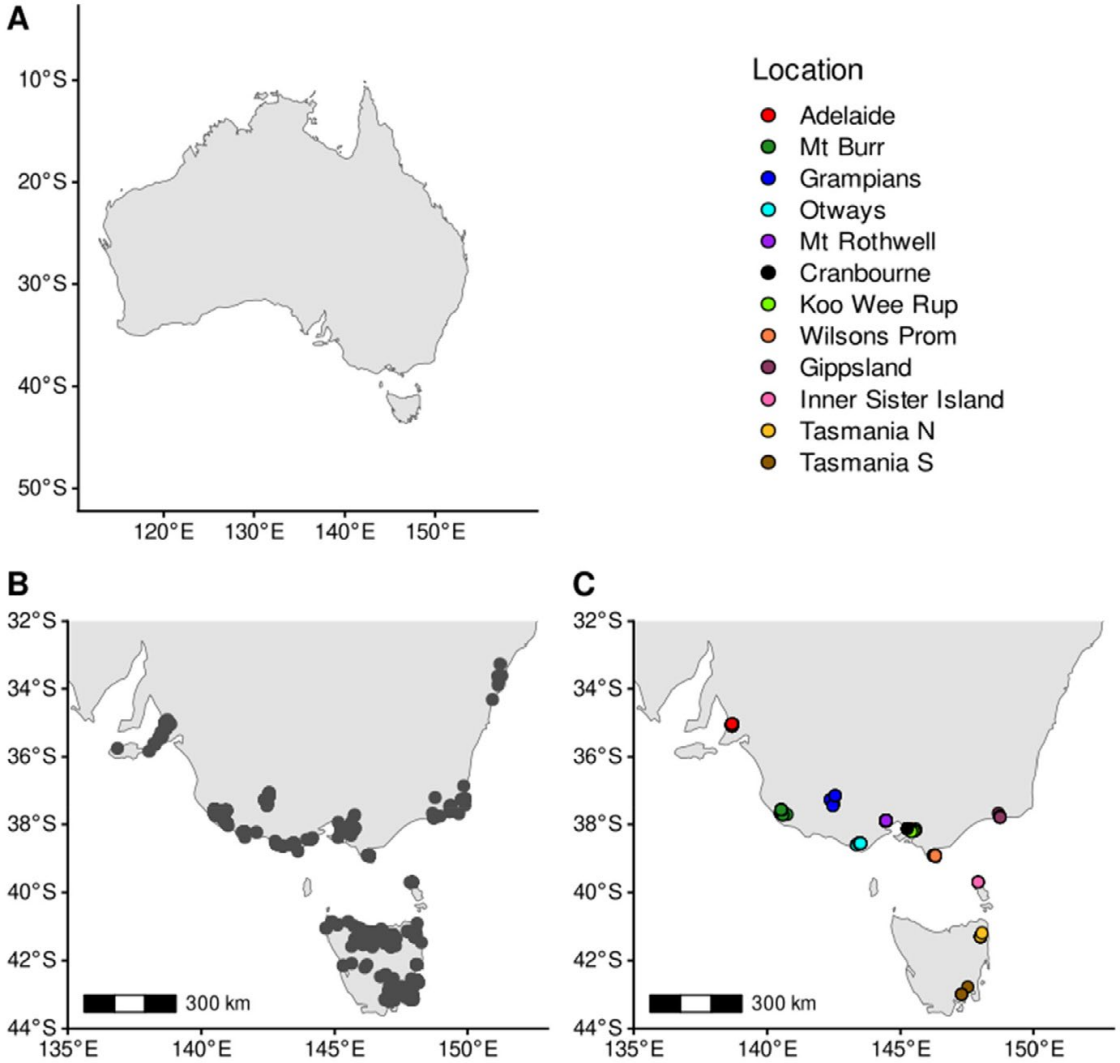
(A) Australia for reference. (B) Public wild distribution records, sourced from the Atlas of Living Australia, and (C) sample locations for *Isoodon obesulus* samples across south‐eastern Australia. Populations and sample sizes after filtering were: Adelaide Hills (*n* = 19, hence Adelaide), Mt. Rothwell Biodiversity Interpretation Centre (*n* = 21, hence Mt. Rothwell), Mt. Burr (*n* = 15), Grampians (*n* = 9), Greater Otways NP (*n* = 10), Cranbourne Royal Botanic Gardens (*n* = 21, hence Cranbourne), Koo Wee Rup swamp (*n* = 14), Wilsons Promontory NP (*n* = 6), Gippsland (specifically Cape Conran, *n* = 5), Inner Sister Island (*n* = 4), Tasmania North (*n* = 9), and Tasmania South (*n* = 4).

Tissue samples were taken via 2‐mm ear biopsy from wild *I. obesulus* populations across Victoria, Australia, through trapping under the Victorian Department of Environment, Land, Water, and Planning animal ethics permit 10009611, and additional South Australian, Tasmanian, and some Victorian ear tissue samples were provided on request by researchers and museums. We obtained 183 samples, dated from 2008 to 2023. All samples were extracted with a Qiagen DNEasy Blood and Tissue kit according to manufacturer's protocol and sent to Diversity Arrays Technology, University of Canberra, for DArTseq genotyping (Cruz, Kilian, and Dierig 2013; Kilian et al. 2012). Data were received as raw reads in fastq format. All samples had metadata or coordinates allowing for a location precise to within 1 km, except for 19 Tasmanian samples that had only a nearest town recorded. Samples were grouped into 12 populations based on known geographic boundaries (Figure 1). Sex data were only available for 61 female and 71 male animals; however, this is more than sufficient to screen sex‐biased loci (Figure S1).

### 2.2 Sample and Genotype Filtering

FASTQ raw data files were demultiplexed using the command *process_radtags* from the Stacks2 (v2.65) suite with the additional settings “‐c ‐q ‐r ‐s 20” (a minimum rolling PHRED score of 20) to discard low‐quality reads. Samples had 5′ adapters and Illumina universal sequencing adapters removed using *cutadapt* v3.4 (Martin 2011). We trimmed longer reads to 80 bp using *trimmomatic* v0.39 (Bolger, Lohse, and Usadel 2014) and discarded any reads below 80 bp using *fastp* v0.12.4 (Chen et al. 2018). We then aligned samples *de novo* using the pipeline in Stacks2 “denovo_pl.sh” v2.65 (Rochette, Rivera‐Colón, and Catchen 2019), assessing the number of allowed mismatches (−*M* and −*n* parameters) of 1–6, and −*M* = −n, as per Rivera‐Colón and Catchen (2022). The maximum number of SNPs for our dataset was found to be at −*M* = −*n* = 1, corresponding to 397,700 sites (Table S1). Once optimal −*M* and −*n* parameters were determined, we used this run of the *de novo* pipeline for downstream analysis, First, we screened our samples for missing data using *populations* v2.65 from the Stacks2 package, with the parameter −*R* = 0.8. Per‐sample missingness was reported using *vcftools* v0.1.16 (Danecek et al. 2011). We selected a threshold of < 30% missing data per sample, with 171 samples retained at this threshold. A threshold of < 10% would have resulted in 162 samples retained, but critically the Wilsons Promontory NP (hence Wilsons Prom) population would have had its sample size reduced to five. *gstacks* v2.65 was rerun on retained samples to form a new catalogue. *Populations* was then rerun for these sample using the following parameters: “‐R 0.8, ‐r 0.8, ‐p 11, ‐‐min‐mac 3.” Loci were further screened by read depth using *vcftools*, according to Li's maximum read depth (Li 2014), resulting in initial parameters of ‐‐min‐meanDP 5 and ‐‐max‐meanDP 67.

Samples were assessed for sex‐biased loci using “gl.report.sexlinked” from the R package *dartR* v2.9.7 (Mijangos et al. 2022); we detected no sex‐biased loci (Figure S1). We then assessed samples for kinship using estimates of Loiselle's *K* (Loiselle et al. 1995), calculated by *Spagedi* v1.5 (Hardy and Vekemans 2002). We removed probable full siblings or parent–offspring to leave no more than two animals with first‐degree kinship group, as per Waples and Anderson (2017), defining first‐degree kinships as *K* > 0.18. This resulted in the removal of 34 samples, with a final dataset of 137 samples (Figure 1). The dataset was rescreened for Li's maximum read depth, resulting in final filtering of loci by mean depth between 5 and 71, and 7333 SNPs. *TreeMix* and *conStruct* require the removal of linked SNPs; therefore, the input files were thinned to only include loci outside of 10,000 base pairs of each other using *vcftools* “‐‐thin 10,000.” This limited each RADtag to a single SNP, resulting in 5221 SNPs for these analyses. Downstream data analysis did not indicate any batch effects arising from separate DArTseq plates.

### 2.3 Genetic Structure, Gene Flow, and Genetic Diversity

To visualise genetic structure among individuals, we generated a principal component analysis (PCA) of pairwise genetic differences in R v4.2.3 and RStudio v2022.07.2 (R Core Team 2021; RStudio Team 2020) using the packages *stats* (R Core Team 2021) and *ggplot2* v3.4.2 (Wickham 2016). The Adelaide Hills population has previously been reported to be highly divergent, and Mt. Rothwell Biodiversity Interpretation Centre (hence “Mt. Rothwell”) is a fenced site established by translocations from Adelaide populations, so we created a second, reduced dataset with these populations removed for analyses of genetic differences within Victoria and Tasmania.

We used *fineRADstructure* v0.3.1 and *Radpainter* v0.3.1 to estimate shared coancestry between individuals by creating a heatmap and dendrogram of haplotype similarity (Malinsky et al. 2018). We used *conStruct* v1.0.5 (Bradburd, Coop, and Ralph 2018) to compare clustering models that did or did not incorporate a spatial decay component into sorting. *conStruct* employs a non‐spatial model that uses genetic clustering for a specified value of *K* clusters and a spatial model where discrete clustering occurs alongside a continuous spatial decay of genotype similarity. If population structure can be explained by uniform spatial factors such as distance, then this structure can be presented with a single genetic cluster. *K*, the number of background clusters, was selected using *conStruct's* cross‐validation tool, at 4 repeats of 500 iterations for each value of *K* from 1 to 7 under spatial and non‐spatial models. After *K* selection, *conStruct* spatial and non‐spatial models were run for 5000 iterations each. *conStruct* was run without the Mt. Rothwell population, as this population was established via translocation from another location. Maximum likelihood analyses of ancestral migration and genetic drift of ancestral populations were estimated with *TreeMix* v1.13 (Pickrell and Pritchard 2012), using the Adelaide Hills population as the root. *TreeMix* was run for 100 replicates for each of 0–3 migration fronts, and log‐likelihood of each run reported. Graphs for the highest five log‐likelihood outputs for each number of migration fronts were observed for consistency and ecological soundness. Finally, to assess genetic differentiation between populations, we estimated private alleles with *populations* from Stacks2, pairwise fixed allelic differences using the *dartR* function “gl.fixed.diff,” and pairwise genetic distance (*F*_ST_) using the package *StAMPP* v1.6.3 (Pembleton, Cogan, and Forster 2013). Statistical significance of *F*_ST_ values was assessed by bootstrapping (5000 bootstraps) with a Bonferroni correction at the table‐wide *α*′ = 0.01 level for multiple comparisons.

### 2.4 Simulated Introductions

Using the un‐thinned SNP dataset, we performed simulated introductions using a methodology adapted and modified from Weeks, Stoklosa, and Hoffmann (2016). This code simulates the mixing of a specified number of samples from two populations, in this case a smaller donor population and larger recipient population. Under the assumption that all loci are neutral, the program simulates a new panmictic population between these samples, with the subsequent generations' census size growing by a user‐defined rate each generation. This occurs several times, until the simulation reached a defined maximum population size, and this ends the pre‐mixing step. Then, the simulated population continues panmixia between samples with discrete, non‐overlapping generations, but now the census size of each generation is held stable at the user‐defined maximum population size. The model reports mean heterozygosity at each generation, which will change due to genetic drift over the subsequent generations, as mutation and recombination are assumed to be negligible for this model.

Cranbourne Royal Botanic Gardens (hence “Cranbourne”) was selected to represent the recipient population for simulations, for several reasons. Firstly, it is a fenced population with low autosomal heterozygosity (Figure 7), making it a potential future candidate for genetic management. We can also more accurately parametrise maximum carrying capacity, with Cranbourne having an estimated 300–500 animals depending on seasonal conditions, based on the conservation manager's estimate of appropriate habitat and historical trends at the location (Terry Coates pers. comm.). This reflects literature on *I. obesulus* density estimates of around 2 animals per hectare (Pentland 1999; Ramalho et al. 2018). Finally, as we elected to run the pipeline at a mixing ratio of five recipient population animals per 1 donor population animal, detailed below, having > 20 samples from the Cranbourne population reduces stochasticity by allowing us to simulate an initial mix of 20 recipient to 4 donor samples, a ratio not possible for many of the populations with smaller sample size. For example, Inner Sister Island also represents a population with low heterozygosity (Figure 7) and a fixed carrying capacity but had only four samples, and was therefore deemed unacceptable for simulation despite representing a good candidate.

Five replicate simulated introductions were performed for each donor population scenario, including one scenario where Cranbourne was used as a self‐donor to simulate a non‐introduction scenario. Each introduction was a mixture of 20 randomly selected Cranbourne resident animals and 4 randomly selected animals from each donor population. A 20% addition rate was selected to reflect a “real‐world” event, designed to minimise the risk of genetic swamping of alleles already adapted to the recipient population's location (Fitzpatrick and Reid 2019; Polechová 2022), while still providing sufficient individuals and alleles to avoid genetic founder effects and would facilitate both adaptive and purifying selection to act upon novel alleles introduced (although selection is not simulated in this model). An introduction totalling only 10% of the recipient population was effective in recovering both population numbers and genetic diversity in Burramys parvus (Broom, 1896) (Weeks et al. 2017).

We defined a growth rate (*R*_max_) of 2, so, due to non‐overlapping generations, the initial 24 samples were fully replaced with 48 simulated samples, with each simulated offspring generated from two randomly selected parental genotypes. This estimated *R*_max_ is based on the captive breeding potential of another peramelid, the Eastern barred bandicoot (*Perameles gunnii*, Gray 1838), which are also observed to produce approximately 3 litters of, on average, 2.5 offspring per breeding season, with approximately 30% juvenile recruitment (Black 2020; Winnard and Coulson 2008). Other studies have estimated *I. obesulus* *R*_max_ at 1.31 based on mark‐recapture data in a fire‐affected landscape (Ramalho et al. 2018), and 2.02 based on female age of first reproduction and annual fecundity (Hone, Duncan, and Forsyth 2010). Simulated population growth continued until the population reached our user‐defined ceiling of 300 animals, justified above, which then represented “generation 0.” Following this, each generation totally replaces the previous generation with 300 new animals, generated through panmixia of the previous generation. The population was simulated for 50 non‐overlapping generations, and variant site heterozygosity was reported at each generation. For each population, mean variant heterozygosity across all five simulations and 95% confidence intervals were visualised using *ggplot2*. The code used to produce simulations is available at: https://github.com/jblack222/SimIntro.

Following the simulations, we compared the final observed heterozygosity at generations 50 (hence “simulated Ho”) to several metrics of genetic diversity and differentiation. We used the values for pairwise fixed differences and *F*_ST_ between the recipient population and each donor population as generated above, the initial observed heterozygosity of the donor populations, and the autosomal heterozygosity of the donor populations (as detailed in the following section). Additionally, pairwise estimates of Jost's *D*_est_ were generated by the package *mmod* v1.3.3 (Winter 2012) using the function “pairwise_D” as a metric of absolute unshared variation between the recipient population and donor populations, and unshared allelic richness was generated as q0 D beta diversity (Sherwin et al. 2017) by *dartR* using the function “gl.report diversity.”

### 2.5 Individualised Autosomal Heterozygosity

We assessed autosomal heterozygosity using an individualised autosomal heterozygosity pipeline, based on the pipeline from Schmidt, Thia, and Hoffmann (2024), but modified to process individual samples separately. This provides two key benefits, allowing for unequal sample sizes between groups and using a pseudo‐consensus sequence to accommodate the lack of a true reference sequence for *I. obesulus*. In comparison to variant site (SNP) heterozygosity, autosomal heterozygosity is less biased by sample size, and results are more comparable across populations and studies (Schmidt et al. 2021).

The Schmidt, Thia, and Hoffmann (2024) pipeline takes a VCF file of all samples, genotyped against a reference genome, and then separates individual samples from the VCF for filtering and heterozygosity calculations. This presented three problems when applied to our *de novo* assembled dataset. Firstly, upstream processing of all samples as a batch resulted in population‐level, negative autocorrelation between autosomal heterozygosity and total sites for five of the populations, after a Bonferroni correction at *α*′ = 0.05 for multiple comparisons (Figure S2A). Secondly, due to the use of Stacks2 for *de novo* alignment, genotypes were reported by *populations* which, at time of writing, do not accommodate locus depth information at monomorphic sites, critical for downstream filtering. Separate filtering of individuals by locus depth at all sites, not merely variant sites, is a critical step in the Schmidt, Thia, and Hoffmann (2024) pipeline, as it evades issues of differential missing data (Schmidt et al. 2021), low or differential sequencing depth (Nielsen et al. 2011), excessively high sequencing depth (Li 2014), and polyallelic sites (Sopniewski and Catullo 2024). Finally, and most importantly, filtering at a specific locus is based on a percentage call rate across samples in a group, regardless of the software used for genotyping. This means that the pipeline is sensitive to different sample sizes (Sopniewski and Catullo 2024), and results between groups of different sizes are incomparable. One work‐around is to simply reduce all groups to the minimum sample size; however, when working with small populations this is not always viable. Instead, our solution was to only run single samples through the pipeline before recombining groups downstream, thus bypassing percentile filters.

Calling variant sites after separating individual data is known to limit the inclusion of rare alternate alleles (Koboldt 2020), and increasing distance from a reference genome can result in lower heterozygosity estimates (Duchen and Salamin 2021). However, our objective with this pipeline was to observe the rate of heterozygosity across all confidently genotyped sites (variant and otherwise), rather than to accurately call every low‐frequency variant which may in any case be removed by downstream filtering. Increasing divergence of samples from a reference genome can also result in bias calling variant sites, increasingly for more differentiated samples, which we control for by compiling raw data from all samples to generate the pseudo‐reference sequence. Additionally, the separate processing of individuals from before alignment to the pseudo‐reference sequence makes this pipeline robust to the inclusion or exclusion of samples, without changing heterozygosity estimates for any sample.

### 2.6 Running the Pipeline

**FIGURE 2:**
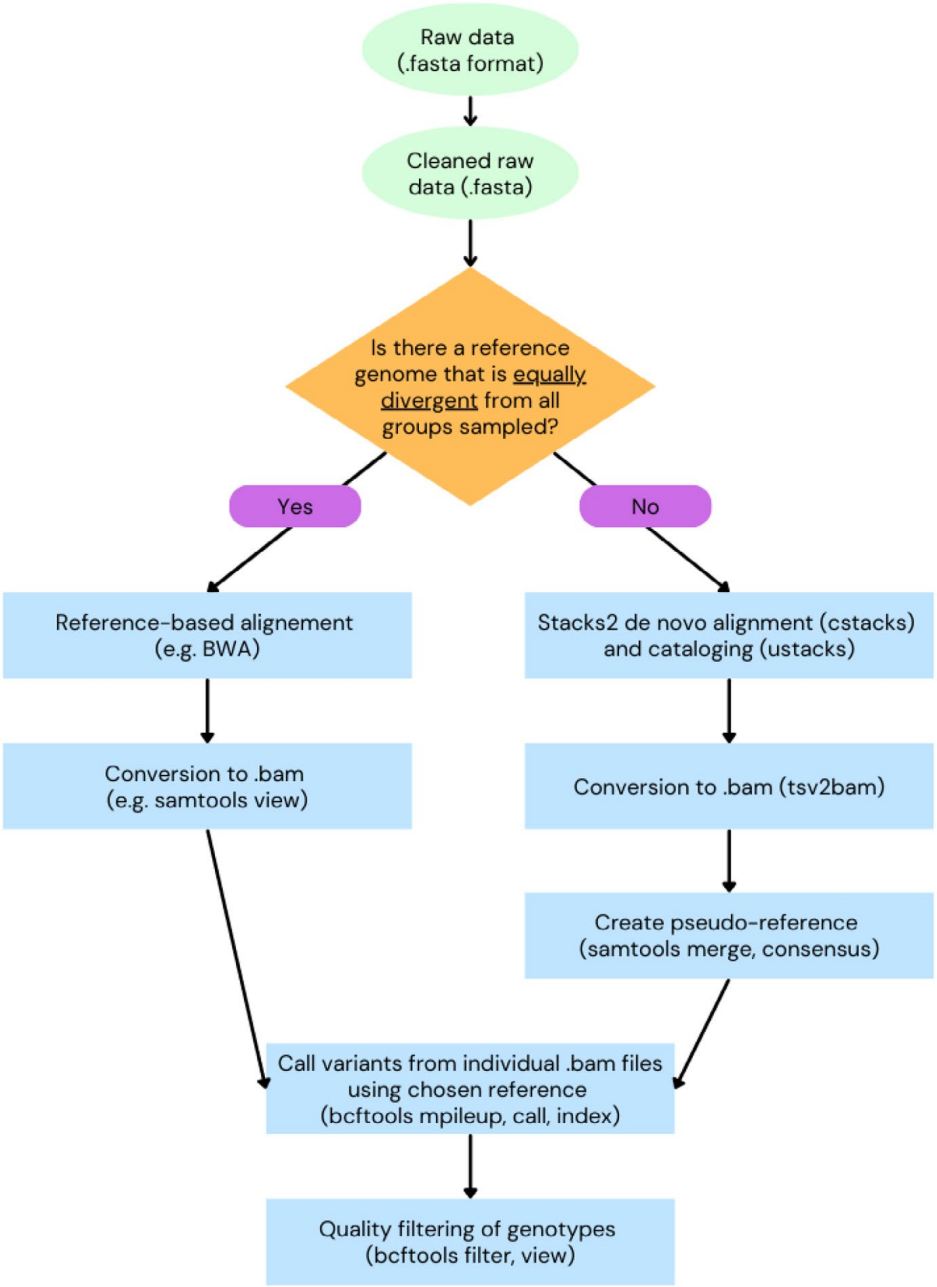
Overview of programs used to estimate individualised autosomal heterozygosity, using either reference‐based and de novo alignment.

Raw data in FASTQ format are first adaptor‐cleaned and trimmed to uniform length, as for our previous analyses. For de novo, raw data are then passed to the Stacks2 *cstacks* and *ustacks*, which run de novo alignment and cataloging. Then, we use *tsv2bam* to generate individual sample BAM files and process these using *samtools* v1.18 (Danecek et al. 2021). We use *samtools merge* to create a merged BAM file from all individual samples output by *tsv2bam* and then used *samtools consensus* on the merged BAM file to generate a FASTA pseudo‐reference assembly. This pseudo‐reference sequence is representative of all samples, which alleviates genotyping bias arising from varying levels of sample differentiation from the reference sequence (Duchen and Salamin 2021). If a reference genome that is equally representative of all groups is available, reference‐based alignment and conversion of files to BAM format can occur in the traditional way, and a pseudo‐reference sequence is not required (Figure 2). Whether using de novo or reference‐based upstream processing, individual sample BAM files are then independently aligned to the (pseudo‐)reference using *bcftools* v1.18 *mpileup* (Danecek et al. 2021; Li 2011) with the options “‐a FORMAT/DP, ‐a FORMAT/AD, ‐I,” which assign an individual sample locus depth and allele depth to every locus (including monomorphic sites), while removing indels. This produced a processed BCF file for each sample, which was then converted into a final VCF file using *bcftools call* with the option “‐m” for multiallelic calling (Sopniewski and Catullo 2024).

### 2.7 Sample Filtering

Individual sample VCF files were then filtered by *bcftools view* piped into *bcftools filter* to only retain confidently genotyped loci (Figure 2). The parameters for defining “confident” can be flexible to the dataset of the user and adjusted as necessary, but should contain the filtering for the following: maximum locus depth (here 71× (Li 2014), −e ‘FORMAT/DP > 71’), minimum locus depth (here 15× (Nielsen et al. 2011), −e ‘FORMAT/DP < 15’), removal of star alleles (−e ‘ALT = “*” ‘), removal of all loci with missing genotypes for that sample (−e ‘GT = “mis”‘), removal of loci with a quality score below a threshold (here 25, −e ‘QUAL < 25′), and removal of heterozygous sites where either allele had a depth below 3 (−e ‘FORMAT/AD[*:0] < 3′, −e ‘FORMAT/AD[*:1] < 3′). Finally, atomisation of polyallelic sites was performed using *bcftools norm* (−a ‐‐atom‐overlaps (Schmidt et al. 2021)). Each sample is then output into a final VCF file, and heterozygosity across all retained sites (individual autosomal heterozygosity) is calculated.

Autosomal heterozygosity estimates from this pipeline were evaluated for significant differences between populations with ANOVA (Girden 1992) and Tukey's test for honestly significant differences (Sokal and Rohlf 1994), and visualised in R using *ggplot2* and p‐values expressed using *pheatmap* (unpublished). We reassessed population‐level autocorrelation and found that no correlations remained between autosomal heterozygosity and sites called (Figure S2B). The individual autosomal heterozygosity pipeline is available at: https://github.com/jblack222/AutoHet.

## 3 Results

**FIGURE 3:**
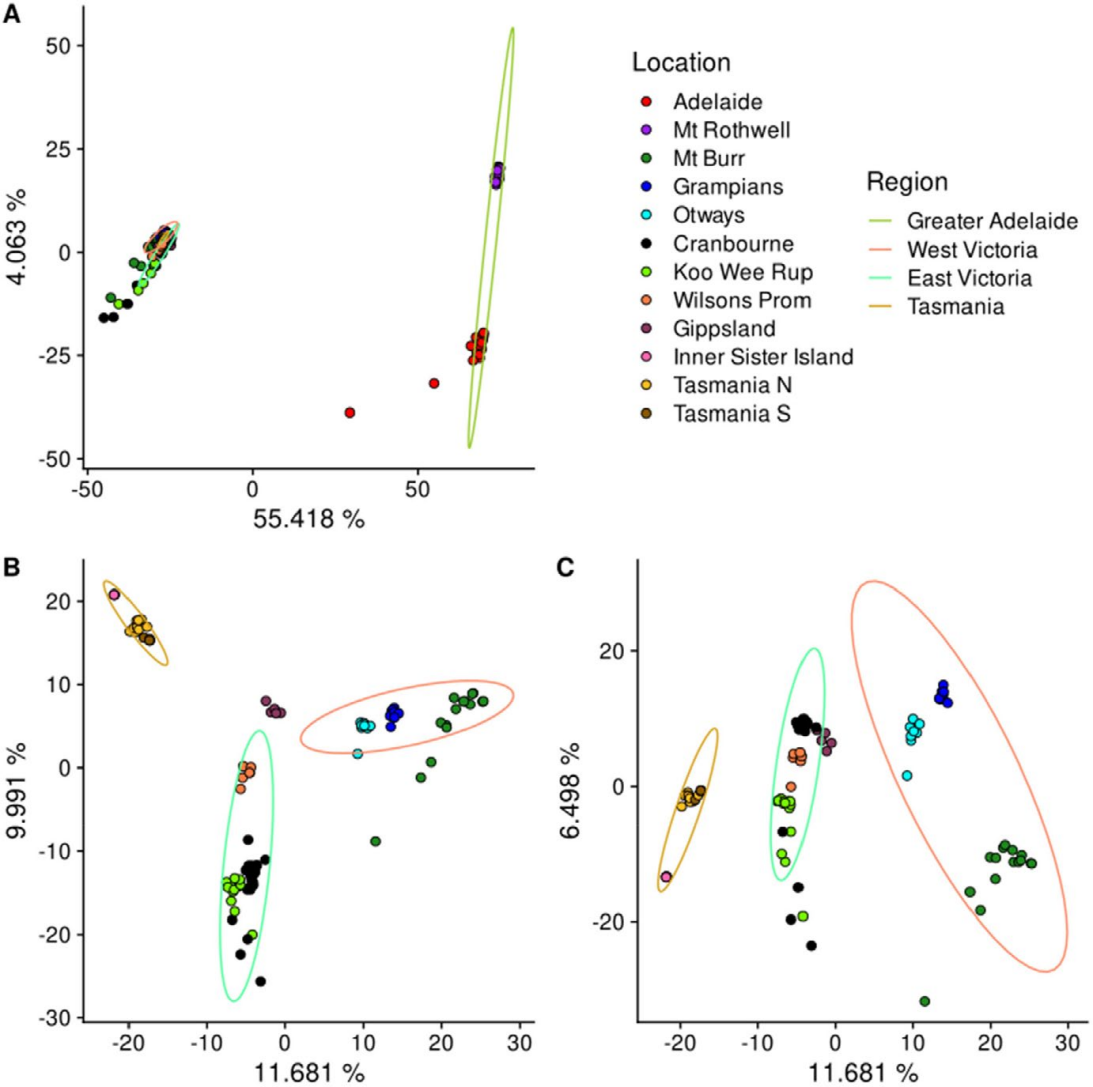
Principal components analysis (PCA) of (A) a full dataset of 137 *Isoodon obesulus* showing components 1 versus 2, and (B, C) a reduced dataset of 113 *I. obesulus* with Adelaide Hills and Mt. Rothwell populations removed, showing components 1 versus 2 and 1 versus 3, respectively. Percentage of variation explained is shown on axis labels.

Strong differentiation was observed between Adelaide Hills and Mt. Rothwell, deemed the “Greater Adelaide” region, and all other sampled locations (Figures 3A and 5A, Figure S3), consistent with previous literature (Cooper et al. 2018; Li et al. 2014; Robinson et al. 2021), and so a reduced dataset was generated with these populations removed to better understand the dynamics of other regions.

**FIGURE 4:**
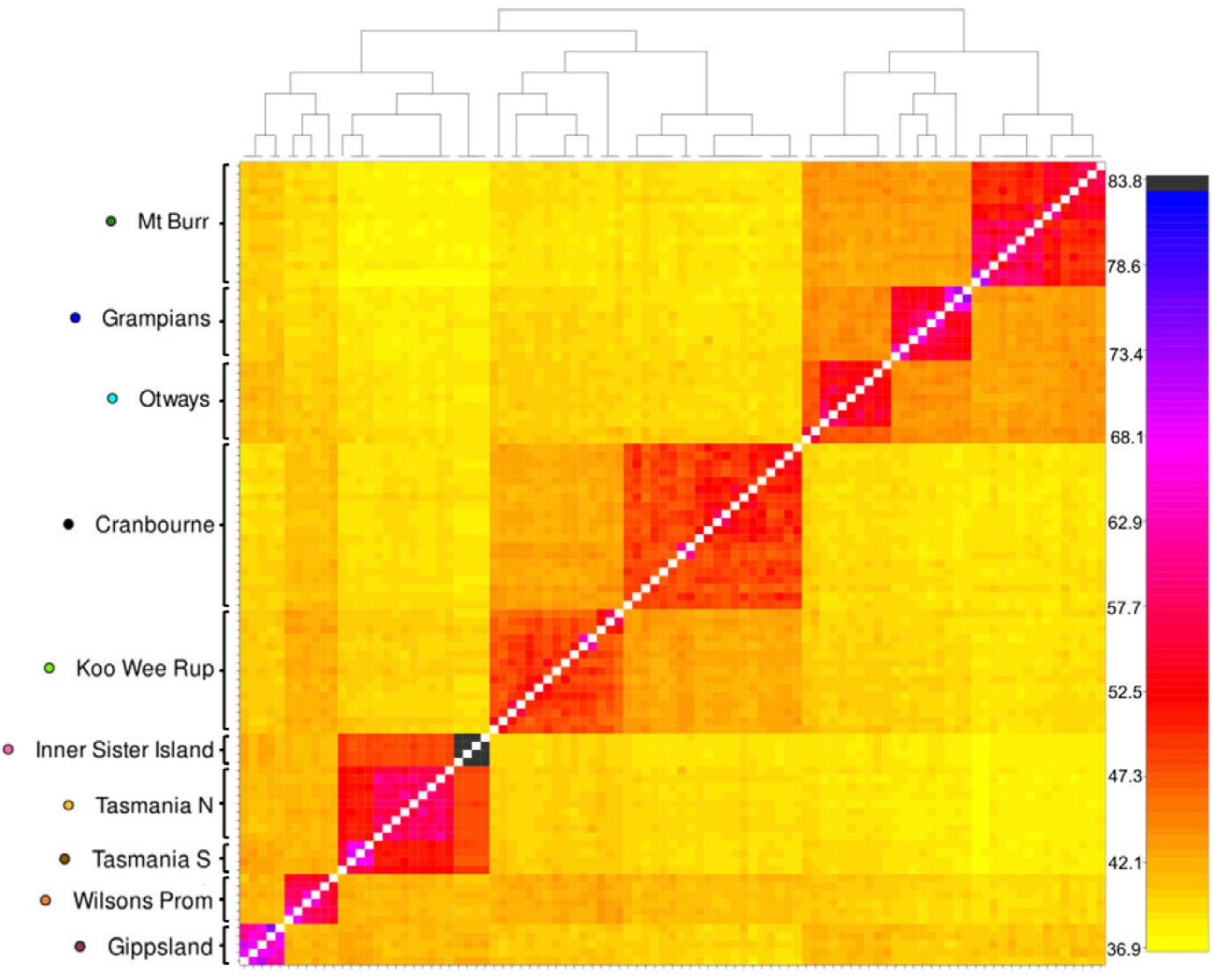
Heatmap of individual pairwise coancestry coefficients generated by *fineRADstructure* in a reduced dataset of 113 *Isoodon obesulus*. Cells indicate degree of pairwise haplotype similarity between individuals and are shaded from yellow (low) to blue (high) to indicate increasing levels of coancestry. Above, a dendrogram of individual clustering based on coancestry coefficients with posterior probability on arms, all arms > 0.87. Left, a priori population assignments indicating group assignment of individuals; no individuals were sorted outside of their a priori population. Analysis with all individuals available in Figure S3.

The reduced dataset showed clear grouping of populations under our a priori assignments, with most showing separate grouping across the first three components of a PCA (Figure 3B,C), and high levels of genetic coancestry of individuals within these population assignments were detected with fineRADstructure (Figure 4). Furthermore, three landscape‐scale regions were identified in the reduced dataset on PCA (Figure 3B,C) and fineRADstructure (Figure 4), with western and eastern Victoria and Tasmanian populations all appearing to be approximately equidistant. The exception was northern and southern Tasmanian samples, which appeared to show substantially lower differentiation than observed between locations separated by similar distances in Victoria, such as Koo Wee Rup and Wilsons Promontory or Mt. Burr and the Grampians (Figures 3 and 4). No additional structure was observed on other principal components. Population substructure was evident for samples from several locations, including the Grampians, the Otways, Koo Wee Rup, and, to a lesser extent, Mt. Burr (Figure 4), and also within the Adelaide Hills cluster (Figure S3). Haplotype similarity of Mt. Rothwell samples was not particularly higher with any single region of Adelaide Hills. High levels of shared coancestry were observed within Inner Sister Island, Gippsland, and the nothern and southern Tasmanian populations. The fineRADstructure similarity dendrogram suggests an initial differentiation between the western and eastern Victoria populations occurring before differentiation between eastern Victoria and Tasmania, with the final connection likely to be either via Gippsland or Wilsons Prom.

With the identification of four landscape‐scale regions, of which one was substantially more divergent than the other three, we next aimed to determine whether this differentiation is driven by spatial factors such as isolation by distance or factors beyond spatial separation alone suggesting additional migration barriers. *conStruct* was used to assess whether the discrete clustering of genetic similarity in these populations was better explained under a spatial model or non‐spatial model.

**FIGURE 5:**
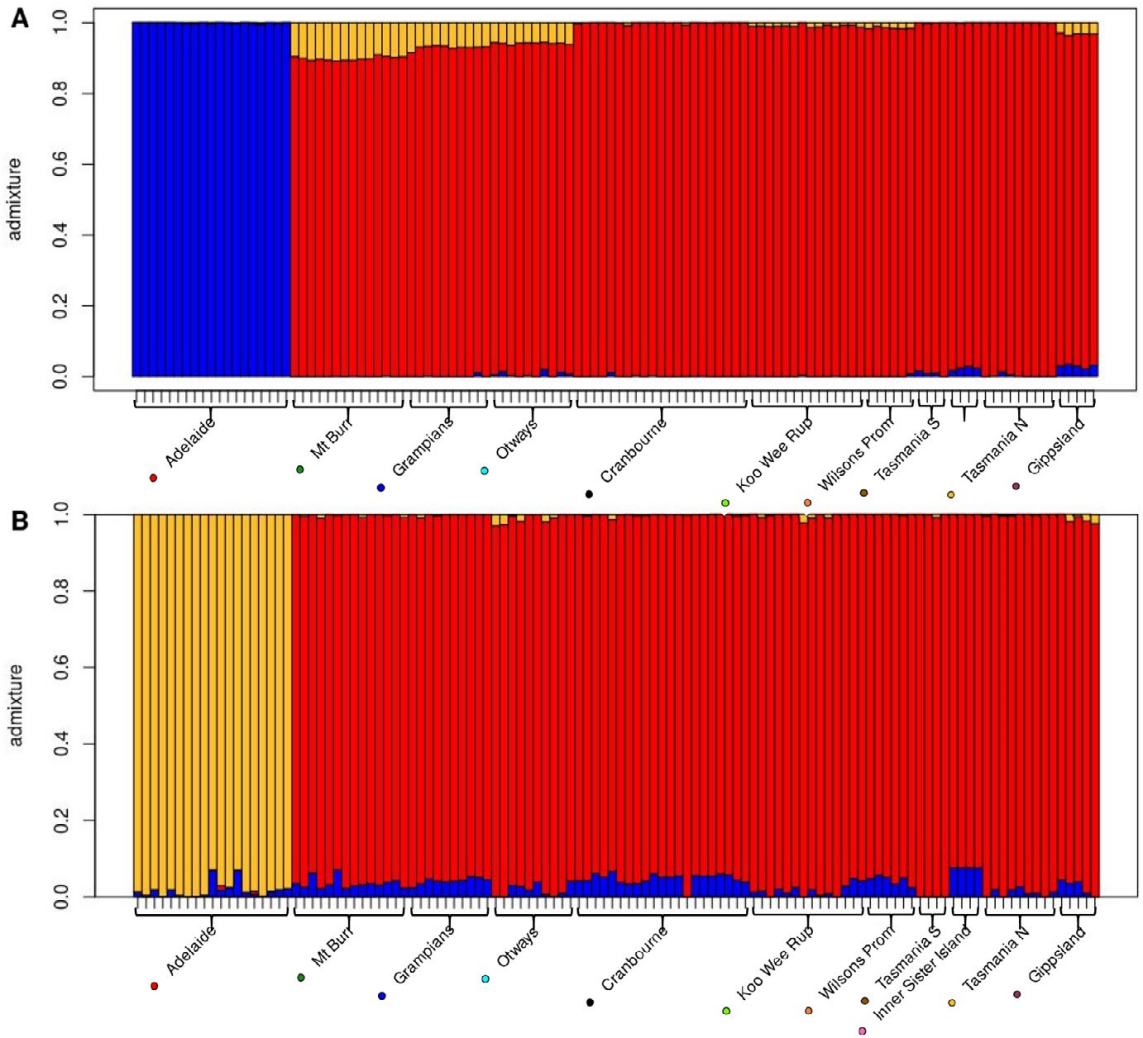
Admixture barplots of *conStruct* non‐spatial (A) and spatial (B) models of genetic clustering in 116 *Isoodon obesulus* in southeast Australia. Samples sorted by longitude from left (west) to right (east). The Mt. Rothwell population is not included, as animals at this location are known to be translocated.

*conStruct* cross‐validation suggested *K* = 3 was appropriate for further analysis, as this represented high predictive power without risking overfitting (Figure S4). At *K* = 3, the non‐spatial model showed a small genetic difference between western and eastern Victoria, and between Victoria and Tasmania, but high differentiation between Adelaide Hills and all other populations (Figure 5A). When a spatial decay component was incorporated, Victorian and Tasmanian samples became more uniform in their clustering, indicating that genetic differences are likely driven by spatial factors, while Adelaide Hills still showed variation not explained by spatial factors alone, suggesting that there are further barriers to gene flow between itself and Mt. Burr (Figure 5B). When removing the Adelaide Hills population from this analysis, no additional spatial or non‐spatial structures were detected, and Tasmanian and Victorian population differences were still accounted for by spatial factors.

**FIGURE 6:**
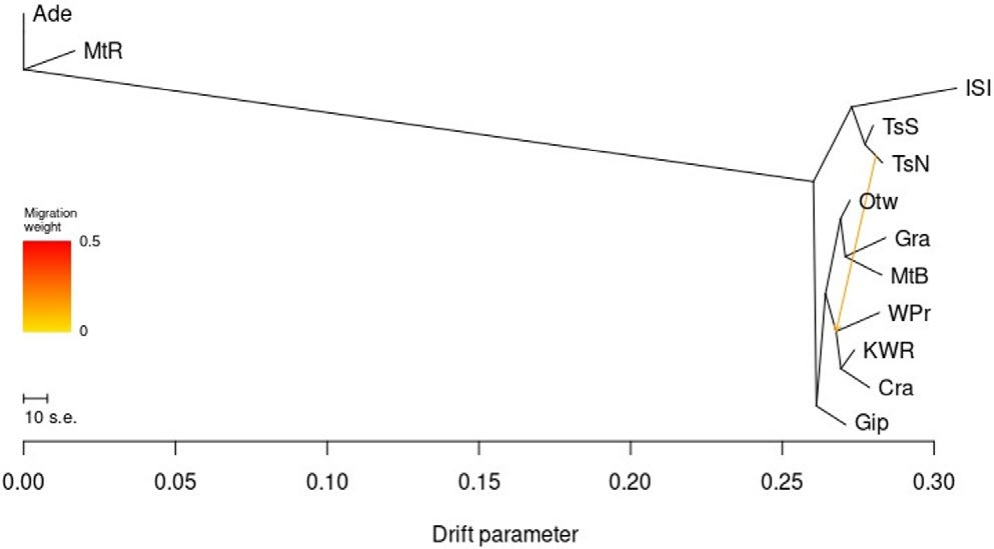
*TreeMix* dendrogram of relative drift between populations of 137 *Isoodon obesulus* across south‐eastern Australia, with Adelaide Hills assigned the root and 1 migration front allowed. Population abbreviations: Ade = Adelaide, MtR = Mt. Rothwell, ISI = Inner Sister Island, TsS = Tasmania South, TsN = Tasmania North, Otw = Otways, Gra = Grampians, MtB = Mt. Burr, WPr = Wilsons Promontory, KWR = Koo Wee Rup, Cra = Cranbourne, Gip = Gippsland.

Minimal migration was observed by *TreeMix* between populations, and high log‐likelihood *TreeMix* outputs with one migration front commonly showed a split between Tasmanian and Victorian populations as the first, and therefore most ancestral, separation after separation from the root population and Mt. Rothwell (Figure 6). This was shortly followed by separation of Gippsland from all other populations, occurring before the divergence of eastern and western Victorian populations, and divergence of populations within Tasmania, with a weak migration front placed between one of the Tasmanian nodes and either a Gippsland or Wilsons Promontory node (Figure 6). When no migration front was allowed (Figure S5), all high log‐likelihood outputs indicated initial separation of Gippsland, followed by Tasmanian, and then eastern and western Victorian populations.

The Adelaide Hills and Mt. Rothwell populations had predictably large pairwise *F*_ST_ and a large number of fixed differences with the other populations, and a low *F*_ST_ and zero fixed differences with each other (Table 1). Similarly, the pairwise *F*_ST_ of the other populations mirrored differentiation seen in previous analyses, with geographically more proximal populations exhibiting lower values, while fixed differences between populations were minimal.

**TABLE 1:**
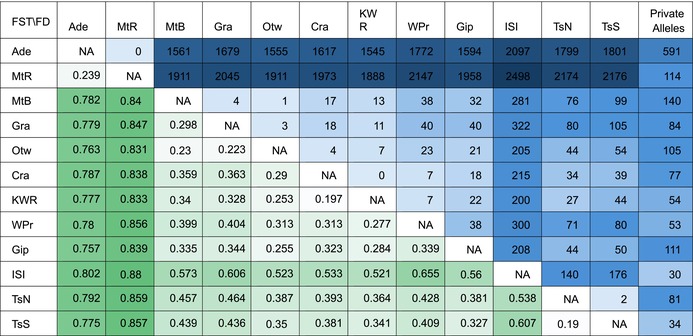
Weir–Cockerham pairwise *F*_ST_ (left of the diagonal), pairwise fixed allelic differences (right of the diagonal), and private alleles (final column) in 137 *Isoodon obesulus* samples.

*Note:* Darker shading indicates larger values. All *F*_ST_ values were statistically significant at the table‐wide *α*′ = 0.001 level after sequential Bonferroni correction for multiple comparisons. Estimates generated with *dartR* (fixed differences), *StAMPP* (*F*_ST_), and *populations* (private alleles). Population abbreviations: Ade—Adelaide, MtR—Mt. Rothwell, MtB—Mt. Burr, Gra—Grampians, Otw—Otways, Cra—Cranbourne, KWR—Koo Wee Rup, WPr—Wilsons Promontory, Gip—Gippsland, ISI—Inner Sister Island, TsN—Tasmania North, TsS—Tasmania South.

### 3.1 Individual Autosomal Heterozygosity

**FIGURE 7:**
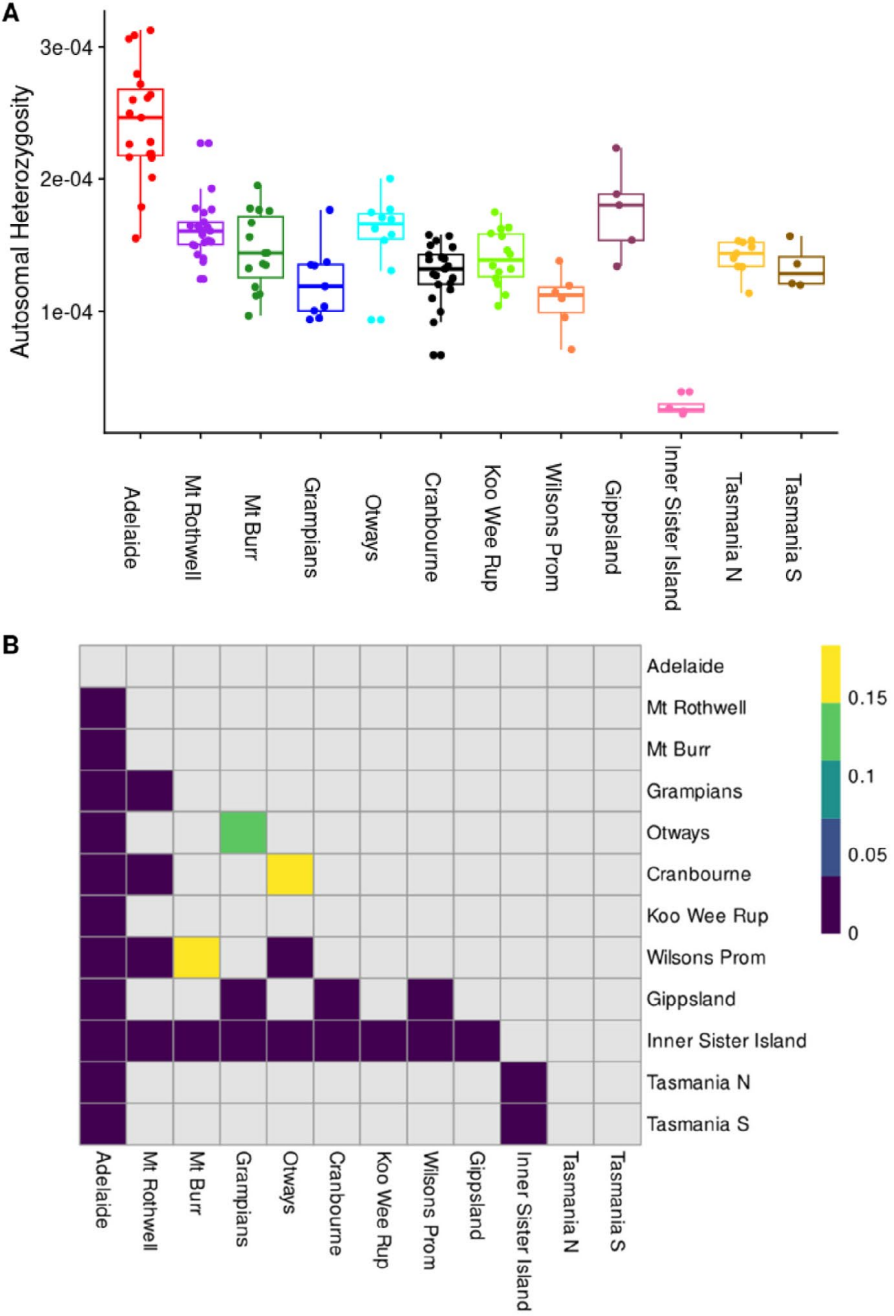
(A) Individual autosomal heterozygosity for 137 *Isoodon obesulus* in south‐eastern Australia. (B) Heatmap of statistically significant pairwise comparisons, as indicated by ANOVA and Tukey's HSD (Table S2). Darkest shading indicates *p* < 0.1 after adjustment for multiple comparisons.

The individual autosomal heterozygosity pipeline produced between 242,649 and 1,283,494 confidently called sites for each sample, with our parameters of maximum locus depth of 71×, minimum locus depth of 15×, quality score threshold of 25, allelic depth of less than 3 for loci called as heterozygous, atomisation of polyallelic loci, and removal of star alleles, spanning deletions, and all loci with missing genotypes. There was no significant correlation between the number of retained sites and autosomal heterozygosity in our populations after Bonferroni correction for multiple comparisons (Figure S2B), with 0 of 12 populations having significant associations at *α* < 0.05, equivalent to a *p* < 0.0041. A one‐way ANOVA indicated significant difference in population means (*p* < 0.0001), and Tukey's honestly significant difference test of multiple comparisons of means indicated several significance groups (Figure 7, Table S2).

Most notably, the lowest heterozygosity population, Inner Sister Island, was significantly lower than all other populations after *p*‐value correction, with all *p* < 0.0001, while Adelaide Hills was significantly higher than all other populations after *p*‐value correction (Figure 7). Other significant groups indicated by Tukey's HSD were that population heterozygosity in Cranbourne, the Grampians, and Wilsons Promontory populations was significantly lower than several other Victorian locations (Figure 7). While lacking in heterozygosity relative to its closely related population of Adelaide, Mt. Rothwell still had a relatively high autosomal heterozygosity compared to all other populations and significantly higher heterozygosity than another captive safe‐haven population at Cranbourne (Figure 7). Variance of individual heterozygosity within all populations was high, with some individuals showing double the heterozygosity of others, while most populations had a limited range of sites reported (Figure 7A, Figure S2B).

### 3.2 Simulated Introductions

**FIGURE 8:**
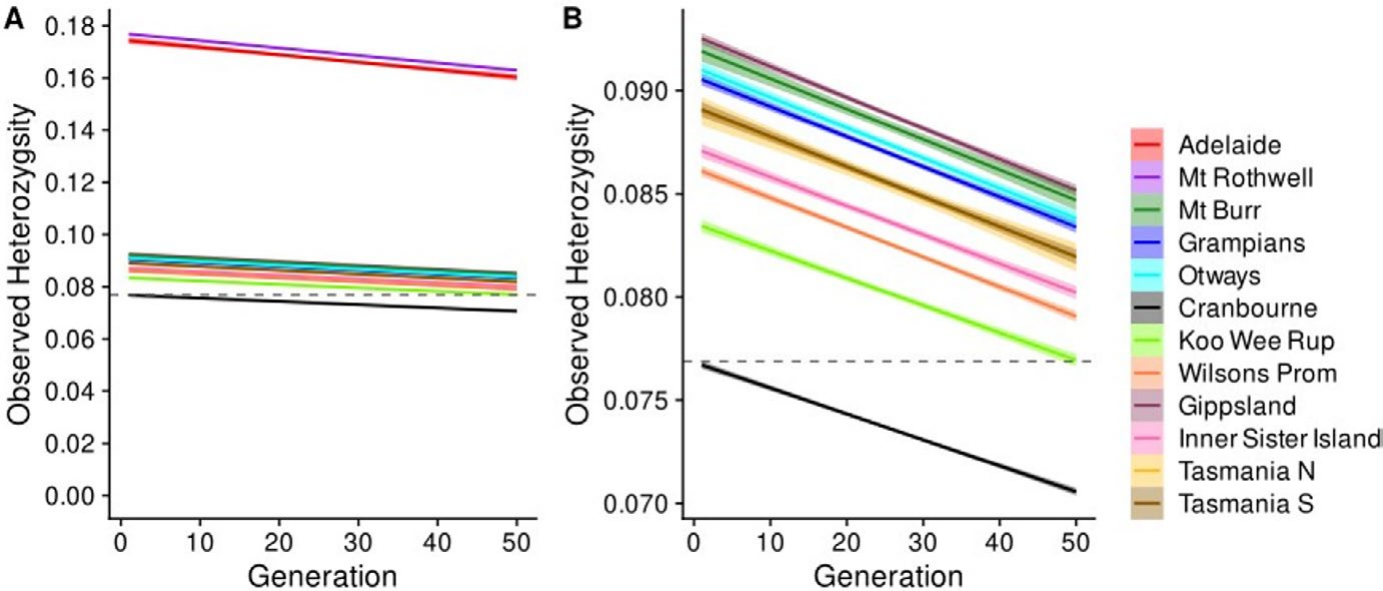
Simulations of population mixing scenarios for *Isoodon obesulus* at Cranbourne Royal Botanic Gardens, tracking change over time of mean observed heterozygosity. For each introduction, 5 simulations in which 4 novel individuals were introduced into a pool of 20 resident individuals, before growth of the population to a ceiling of 300 animals. The subsequent 50 generations are shown. (A) Predictions for all source populations, while (B) shows predictions for the populations for Mt. Burr, Victorian, and Tasmanian source populations only. The dashed line represents Cranbourne Royal Botanic Gardens current observed heterozygosity. The shaded areas around solid lines represent 95% confidence intervals.

Should no mixing occur at Cranbourne, we modelled a small variant site heterozygosity decline of around 8% (between 0.0769 and 0.0707 per simulation) across 50 generations, when compared to the mean initial heterozygosity of the unmixed Cranbourne simulations as indicated by the dashed line (Figure 8). If Cranbourne were to be supplemented by the genetically distant populations of Adelaide Hills and Mt. Rothwell, we model an increase in variant site heterozygosity of 126.8% or 130.0%, respectively, followed by a decline of between 7% and 8% across 50 generations from their simulated maxima (Figure 8). Meanwhile, when observing only the more closely related populations of Victorian and Tasmanian origin, populations geographically proximal to Cranbourne generally provided a smaller increase (Figure 8), only contributing a handful of novel alleles (Table 1), while the more geographically distant populations provided increases between as high as 20.3% (Gippsland, Figure 8), and up to 39 novel alleles (Tasmania South, Table 1). Interestingly, despite exceptionally low autosomal heterozygosity, Inner Sister Island was still modelled to provide a variant site heterozygosity increase of around 13.3% to Cranbourne. Only when supplementing the Cranbourne population with individuals its most proximal neighbour, Koo Wee Rup, was observed heterozygosity projected to drop below the initial level of the unmixed population, albeit over 50 generations. In all simulations, regardless of origin, heterozygosity tended to decline by between 7% and 8% of each simulation's mean initial heterozygosity over 50 generations; this decline is due to the population not being permitted to grow above 300 individuals and represents diversity lost through drift.

**FIGURE 9:**
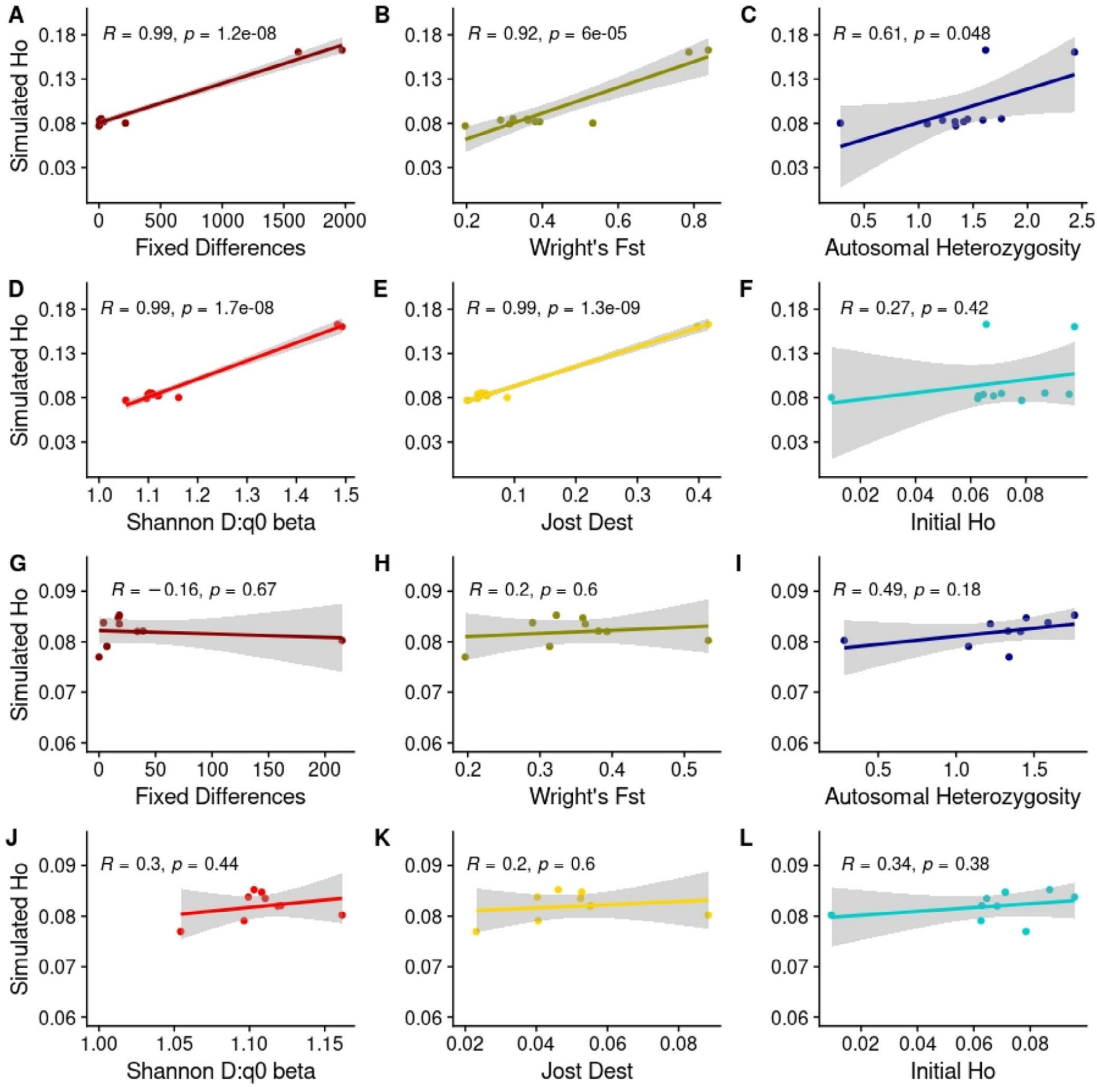
Correlations between final simulated observed heterozygosity (simulated Ho) and six genetic diversity metrics in the full dataset (A–F) and reduced dataset (G–L). Pearson correlation coefficients (*R*) and *p*‐values (*p*) superimposed.

The substantially greater increase in heterozygosity when using Adelaide or Mt. Rothwell populations for mixing is likely due to a large number of common, fixed alleles that are differential between these populations and Cranbourne (Table 1), causing these loci to become heterozygous after mixing with substantial levels of novel alleles retained over subsequent generations. We therefore examined several metrics of differentiation to examine whether any provided strong predictive power for the simulated outcomes of mixing. A Pearson correlation between each donor population's simulated change in heterozygosity after 50 generations and pairwise metrics between Cranbourne and each source population was performed; these were the following: fixed allelic differences, Wright's *F*_ST_, Jost's *D*_EST_, and unshared allelic richness calculated as Shannon *D*:q0 beta diversity (according to Sherwin et al. 2017). Two additional correlations were performed, between simulated change in heterozygosity and each source population's autosomal and variant site heterozygosity. Furthermore, these correlations were examined in both the full dataset (Figure 9A–F) and reduced dataset (Figure 9G–L). In the full dataset, all four measures of pairwise differentiation were found to have statistically significant predictive power (Figure 9A–F), with *p*‐values < 0.00001 and correlation coefficients > 0.9, with Wright's *F*_ST_ slightly underperforming other differentiation metrics. Both heterozygosity measures performed substantially worse than differentiation measures (Figure 9C,F). However, these results are strongly influenced by the two genetically distant populations (Adelaide Hills and Mt. Rothwell), and when only closely related populations are observed, the correlation with differentiation metrics becomes weak, with all *p*‐values > 0.43 and correlation coefficients < 0.3 (Figure 9G,H,J,K). Across closer genetic distances, population mean autosomal heterozygosity became a better but imperfect predictor of simulated heterozygosity increase (Correlation coefficient = 0.49, *p*‐value = 0.18, Figure 9I).

## 4 Discussion

*Isoodon obesulus* has previously been found to be highly structured; differentiation between Adelaide Hills and other populations is well described (Cooper et al. 2018; Li et al. 2014; Thavornkanlapachai et al. 2021), along with structure between western Victorian and Mt. Burr populations (Li et al. 2014; Robinson et al. 2021), and population substructure identified at several locations (Li et al. 2016, 2015; Robinson et al. 2021). Here, we analyse six previously unassessed populations and further scrutinise the genetic structure of this taxon at multiple scales, providing novel observations of autosomal differentiation between mainland Australia and Tasmania, and population substructure at the Greater Otways NP. We supplement this with genomic assessments of diversity and simulations of population mixing across various levels of genetic differentiation.

Population mixing simulations were performed using the Cranbourne Royal Botanic Gardens population as a recipient, because this location is fenced and autosomal heterozygosity was significantly lower than several wild populations. Additionally, site‐managers estimated a carrying capacity of around 300–500 animals (Terry Coates pers. comm.), and so the temporal persistence of any increase in genetic diversity would also be of interest for practical application. In practicality, population viability analysis tools, such as Vortex (Lacy 1993), could be used for recipient population selection; however, testing our simulations required selecting a site with a reasonable amount of genetic data and a confident estimate of carrying capacity. Simulations showed that using either Mt. Rothwell or Adelaide Hills as a donor population for genetic mixing provided a substantially greater increase in heterozygosity than other populations, modelled to increase heterozygosity by more than 100%. This suggests that several alleles entering the recipient population are reverting fixed, homozygous loci to heterozygous form, supported by the rates of private alleles and fixed differences between populations (Table 1). As these populations may in fact represent distinct subspecies, concern may be raised around the possibility of outbreeding depression in the undertaking of population mixing (Hedrick and Fredrickson 2010). However, these simulations model a migration totalling only 20% of the resident population, making it more probable that any introduced alleles under purifying selection would be purged, while also providing the opportunity to maintain or fix new alleles under adaptive selection. Where the likelihood and severity of outbreeding depression are uncertain, mixing should be assessed by preliminary test‐crossing of populations in captivity, to evaluate any symptoms of outbreeding depression including changes in mortality, fecundity, and fitness in F1 though F3 offspring (Hedrick and Fredrickson 2010; Black 2020). If a more conservative approach was taken and, instead, a Victorian or Tasmanian population was used as the donor population, all populations were modelled to provide a sustained increase to heterozygosity even after 50 generations, except the most proximal and most genetically similar population of Koo Wee Rup. This even includes the highly depauperate population at Inner Sister Island, reinforcing the value of unique genetic variation. Furthermore, there is some evidence that small populations, particularly populations that are repeatedly bottlenecked, can undergo genetic purging of strongly deleterious alleles, and so Inner Sister Island may in fact represent a lower risk of carrying these alleles to a target population than larger populations (Dussex et al. 2023). However, Inner Sister Island would likely have a higher rate of homozygous, weakly deleterious alleles due to genetic drift and, although these alleles will be initially masked by mixing and may subsequently be purged, could nonetheless be problematic. The greatest risk in considering Inner Sister Island as a source population is likely to be inbred source contributors being outcompeted by resident animals or contributors from other source populations, thus failing to transfer variation unique to Inner Sister Island. However, it is worth noting that such an effect was not observed in another genetically mixed Tasmanian marsupial (McLennan et al. 2020). Changes in heterozygosity after genetic mixing were found to be poorly predicted by differentiation metrics when populations were weakly divergent, but well predicted by differentiation metrics when populations were strongly divergent. When considering highly divergent populations, all measures of genetic differentiation assessed were good predictors of translocation efficacy, and outperformed autosomal and variant site heterozygosity. However, when removing the highly differentiated Adelaide Hills and Mt. Rothwell populations, differentiation metrics became weak predictors of heterozygosity change. This highlights the value of simulations; although this model has limitations, such as the inability to incorporate sex data or simulate overlapping generations, it is still able to better inform mixing decisions than genetic metrics alone, and we encourage other researchers to further develop and refine simulation approaches. Furthermore, results presented in this way are more easily interpreted by conservation decision makers, who do not have to attempt to consolidate multiple metrics they may be unfamiliar with and are instead provided with an explicit comparison of populations for a given scenario. Regardless of the population selected as the donor, a small decline in the heterozygosity of the mixed population occurred due to drift; this was at a consistent rate for all simulations due to the constant, fixed population size. This suggests that a substantial number of novel alleles are retained for many generations, even in a relatively small population, if not purged or fixed by purifying or adaptive selection (i.e., selection that will cause loci in a population to tend towards homozygous state due to conferring a fitness advantage or disadvantage). Thus, a single, well‐executed mixing event may be effective at providing genetic variation over a large number of generations, even when populations are relatively closely related.

The movement of Greater Adelaide animals could be considered a form of genetic provenancing (Hoffmann, Miller, and Weeks 2021), as coastal South Australia is generally considered as “warm temperate” climate, while Victoria is considered “cool temperate.” Provenancing is often employed in extremely low motility organisms, such as plants, where individuals adapted along a cline are unable to spread adaptive alleles ahead of shifting selection pressure. Projected shifting of clines, such as extreme thermal tolerance, due to climate change are increasingly being considered as potential opportunities to implement genetic provenancing proactively (Montwé et al. 2018). More likely, the genetic mixing described here would be better defined as evolutionary rescue (Bell 2017), as the justification for translocation is based on the increased heterozygosity provided, and thus assumes that new alleles will enable adaptation, rather than exhibit pre‐adapted phenotypes. Increases in both individual heterozygosity and novel genetic variation have been implicated in higher fitness and subsequent increases in population size of fragmented, high‐drift populations (Fitzpatrick et al. 2020), similar to some populations of *I. obesulus* described here.

Autosomal heterozygosity is still an emerging technique, and there are ongoing attempts to refine the specific method of calculation (Schmidt, Thia, and Hoffmann 2024; Sopniewski and Catullo 2024). Currently, no genotyping program, in a single step, can process samples in an individualistic manner as done here; however, many prominent genotyping software (such as Stacks2, GATK, and bcftools) have the capacity to include non‐variant sites in heterozygosity calculations and output files, and also calculate heterozygosity for the genomic data of a single individual. These software should consider the introduction of an individualised version of heterozygosity calculation, as this would lead to a significantly smoother processing method than the one described here. Bcftools was selected for this pipeline over Stacks2 and as it easily outputs non‐variant sites into VCF format, and encodes read depth at these sites which is critical for downstream filtering. This represents a significant loss of filtering ability for both grouped and individualistic calculations of heterozygosity and may result in bias due to differences in read depth between samples. These software should implement this information tag expeditiously.

The most critical benefit of the individualistic calculation of autosomal heterozygosity is its resilience to different sample sizes, a benefit that applies to both de novo and reference‐aligned datasets. As shown in Sopniewski and Catullo (2024), autosomal heterozygosity is substantially altered by differential sample size, and mitigation by down‐sampling to the minimum sample size massively reduces the data available. Here, autosomal heterozygosity of the most heterozygous sample in a population was approximately double that of the lowest, which could cause significant volatility in results if down‐sampling to very small numbers. This is particularly relevant to conservation datasets, as often populations are extinct or extremely difficult to sample, resulting in unavoidably low sample numbers. This would force researchers to decide whether to remove samples across several populations or remove smaller populations entirely. With the individualised method, this ultimatum no longer applies. The primary drawback to this method of heterozygosity calculation is the reduced sensitivity to rare variants, due to the globally reduced confidence in genotyping when running singular samples (Koboldt 2020). Rare variants can be critically important to understand whether adaptive or deleterious, and so we strongly recommend running a separate analysis for rare variant detection if assessing functional variants between populations.

Another benefit of this pipeline is the demonstration of a merged pseudo‐genome as a genotyping reference sequence, applicable not only to de novo datasets but also reference‐aligned datasets when some populations are highly divergent from the reference sequence. Using specifically Greater Adelaide or non‐Greater Adelaide samples to form the pseudo‐genome reference sequence biased the heterozygosity of unrepresented populations upward with fewer total sites called (Figure S6). As the pseudo‐reference is generated from all samples, it therefore should be representative of all samples, making it relatively unbiased. The value of a representative reference sequence is likely to be highlighted as pan‐genomic techniques continue to reveal extreme genomic differentiation within species (Gerdol et al. 2020).

The strongest genetic structure was exhibited between animals from Adelaide Hills and the remainder of the *I. obesulus* populations we analysed, reflecting previous observations (Cooper et al. 2018; Li et al. 2014; Robinson et al. 2021). Mt. Rothwell is believed to have been founded by animals from the Adelaide Hills (specifically Warrawong Wildlife Sanctuary, Annette Rypalski pers. comm.), which our data confirm as highly likely. Cooper et al. (2018), among others, suggest that strong genetic differentiation may be indicative of subspecies or even full species delineation of Adelaide Hills animals. However, under a unified species concept (De Queiroz 2007), species delimitation should be justified on several lines of evidence of diverse types, not simply genetic. This may include morphological differences, differential ecological adaptation, or recognition (pre‐zygotic) and reproductive (post‐zygotic) barriers to breeding (Aldhebiani 2018; De Queiroz 2007).

Presently, the only molecular comparison of Victorian and Tasmanian *I. obesulus* is that of Cooper et al. (2018), in which gene sequence paraphyly concluded that both Tasmanian and Victorian populations ought to be part of an “eastern” *I. obesulus* grouping, with Tasmanian animals maintaining sub‐specific status as *I. o. affinis* based on 2.3% sequence divergence in *ND2*. Our genomic data find that Tasmanian *I. obesulus* are similarly differentiated from most Victorian populations as Victorian populations are from each other, with Gippsland *I. obesulus* showing less differentiated from Tasmanian populations than from western Victorian populations. Spatial factors, such as isolation by distance, were found to be equally strong drivers of differentiation within and between Tasmanian and Victorian populations. However, Tasmanian populations still showed clear nuclear differentiation from Victorian populations, and so further lines of evidence would be valuable for supporting or rejecting the subspecies delineation *I. o. affinis*. Similar temporal and nuclear differentiation is reflected in another peramelid, *P. gunnii* (Black et al. 2024; Weeks et al. 2013), which is not reproductively isolated between Victoria and Tasmania (Black 2020).

Genetic differentiation between samples within Tasmania was remarkably low. An analysis of Tasmanian *P. gunnii* found strong structure between northern and southern regions, and fine‐scale structure between 7 populations, of which two showed strong, recent isolation (Black et al. 2024). Although samples were limited, structure in *I. obesulus* appears to be much weaker, more similar to larger Tasmanian terrestrial fauna, such as Tasmanian devils (*Sarcophilus harrisii*, Boitard 1841), eastern quolls (*Dasyurus viverrinus*, Shaw 1800), eastern bettongs (*Bettongia gaimardi*, Desmarest 1882), and long‐nosed potoroos (*Potorous tridactylus*, Kerr 1792), all of which have gene flow in population cores with some isolated regions due to factors unique to each species (Cardoso et al. 2014; Farquharson et al. 2022; Frankham et al. 2016; Miller et al. 2011; Proft et al. 2021). *Isoodpm obesulus* are located more widely in Tasmania than *P. gunnii*; however, the distance between the northern and southern samples, at approximately 200 km, is comparable to the distance between several mainland populations, and a substantially greater distance than between Cranbourne and Koo Wee Rup, where strong genetic structure was found. This is therefore suggestive that ongoing gene flow occurs across Tasmanian *I. obesulus* populations, as it does in other Tasmanian taxa (making *P. gunnii* an interesting exception), with lower levels of fragmentation resulting in lower genetic drift and few fixed alleles between populations.

Inner Sister Island was found to exhibit extremely low autosomal heterozygosity and high internal coancestry, suggesting severe and sustained inbreeding. The four samples from this location were collected in 2010 on a biodiversity survey (Harris and Reid 2011), with the authors inferring *I. obesulus* to be abundant but “considerably smaller” than Tasmanian *I. obesulus*. This may be a result of depauperate genetic diversity or could represent an example of insular dwarfism (Foster 1964; Lokatis and Jeschke 2018) that would imply limited available resources, as was found in island populations of *I. a. barrowensis* (Dunlop and Morris 2018). Inner Sister Island is believed to have had intermittent connection with Tasmania between 14,000 and 6000 years ago, representing substantial time for genetic drift to occur (Adeleye et al. 2021; Harris and Reid 2011). However, approximately two thirds of its area is grasslands or herbfield (Harris and Reid 2011), appropriate habitat for *I. obesulus* in the absence of high predation pressure. *I. obesulus* typically occur at a wild density of around 1–2 bandicoots per hectare in good habitat (Pentland 1999), so at 748 ha Inner Sister Island should have sufficient habitat for a self‐sustaining population of 500–1000 animals (Frankham, Bradshaw, and Brook 2014; Franklin, Allendorf, and Jamieson 2014). The extremely low genetic diversity here is therefore unexpected, and further assessment of its overall health should occur as a matter of urgency. Unfortunately, a small sample size meant that modelling supplementation scenarios, or examining bottlenecks and founder effects for this population was impossible for these samples. Based on the results of the simulated introductions at Cranbourne Royal Botanic Gardens, and the correlation of fixed allelic differences and *F*_ST_ with projected heterozygosity increase, we suggest that supplementation with any animals from South Australia or Victoria would likely result in a substantial increase in genetic diversity.

Population substructure was observed in the Otways, between two proximal areas of heathland known as the Carlise Heath, with exact sample locations between 10 and 13 km apart. Samples were fragmented by agricultural properties around the town of Carlisle River, with eight samples east and two samples south. Both sub‐regions are bordered by sclerophyll forest to the north and wet temperate woodland to the south, both sub‐ideal for *I. obesulus*. While we are somewhat limited in our conclusions by samples size, there is emerging differentiation in haplotypes coancestry between these two habitat patches, suggesting a failure to share genetic material. Agricultural fragmentation is highly unlikely to have occurred before European settlement, so these areas are temporally separated by 250 years at most. This has critical management implications for the Otways, as the Carlisle Heath is a key refuge for a myriad of threatened species, including *Pseudomys novaehollandiae* (Waterhouse, 1843), *Antechinus minimus maritimus* (Geoffroy, 1803), *Mastacomys fuscus* (Thomas, 1882), and *P. tridactylus* (Kerr, 1792) (Magnusdottir et al. 2008; Pla et al. 2023; Shipway et al. 2020; Wilson, Lock, et al. 2017; Wilson, Zhuang‐Griffin, et al. 2017). If animals are unable to migrate between these two sub‐regions, population sizes are effectively halved at both locations, which will result in a greater rate of genetic drift and increased losses of genetic diversity.

## 5 Conclusions

Simulated introductions of *I. obesulus* into Cranbourne Royal Botanic Gardens revealed that introducing animals from source populations across a greater genetic distance provided a fivefold greater increase in observed heterozygosity than more similar populations. However, even closely related populations provided a sustained increase to heterozygosity over 50 generations. Differentiation metrics alone are weak predictors for increases in heterozygosity, so we strongly encourage the development and use of additional analyses such as simulations when selecting source populations. As Victorian *I. obesulus* populations are declining, their evolutionary potential would be enhanced by genetic mixing across the greatest safe genetic distance, and so species delimitation remains an important objective. Genomic differentiation between animals from Adelaide Hills and Mt. Burr was observed, but is insufficient evidence for species delimitation, and non‐genetic evidence for species determination is required. Genomic differences between Tasmania and Victoria are comparable to differences between populations within Victoria, although a population at Inner Sister Island was found to be extremely genetically depauperate, having been separated from the main island of Tasmania for 6000 years, and would likely benefit immensely from genetic mixing. Fragmentation was observed between western and eastern sections of Carlisle Heath in the Greater Otways NP, an area of critical habitat with serious implications for the management of all threatened taxa in this region. Implementation of an individualised autosomal heterozygosity method produced heterozygosity estimates that were unbiased by differential sample size, showing promise for assessing populations with small sample sizes without requiring down‐sampling of larger populations.

## Ethics Statement

Permission to collect tissue samples was provided by DEECA under permit number 10009611 and adhered to all local guidelines.

## Conflicts of Interest

The authors declare no conflicts of interest.

## Benefit Sharing Statement

This project establishes a collaboration between several institutes in Victoria, the South Australian Museum, the University of Adelaide, the Australian Museum, and the Tasmanian Department of Natural Resources and Environment through the acknowledged sample contributors, through whom the manuscript will be disseminated. Insights from this work are being directly implemented in management plans and translocations for *I. obesulus*, overseen by the Victorian Department of Energy, Environment, and Climate Action. Further benefits from this research will also accrue from the public availability and demonstration of our autosomal heterozygosity pipeline, and simulated introduction approach to modelling heterozygosity change, both of which allow researchers to improve analyses commonly used for conservation assessments. Finally, benefits will accrue from the sharing of our DArTseq data and results on public databases, allowing other researchers to include them in future analyses.

## Supporting information


Appendix S1.

Data S1.

## Data Availability

Sample raw genetic data, and location and sex metadata are publicly available on DataDryad, at doi: 10.5061/dryad.34tmpg4v3. Code used to produce autosomal heterozygosity estimates is publicly available on GitHub, at URL: https://github.com/jblack222/AutoHet. Code used to simulate population mixing is publicly available on GitHub, at URL: https://github.com/jblack222/SimIntro.
