# Supplementary material for: Simulating Genetic Mixing in Strongly Structured Populations of the Threatened Southern Brown Bandicoot (Isoodon obesulus)
Source: Evol Appl. 2024 Dec 5;17(12):e70050. doi: 10.1111/eva.70050 (PMC11621039; doi:10.1111/eva.70050)
Supplement: Supplementary file 1 — Appendix S1. [file EVA-17-e70050-s002.docx]

# Appendix S1: Study Species

*Isoodon obesulus* is a terrestrial marsupial that inhabits south-eastern Australia, with populations reaching from Adelaide to Sydney, and found across Tasmania. It is a highly fecund species, with female adults producing approximately 3 litters of between 1-5 offspring across a spring breeding season (Mallick et al., 1998; Stoddart and Braithwaite, 1979). Individuals in captive sites have been observed to live up to 4 years (Black, unpub. data), however typical survival in the wild is likely much lower (Mallick et al., 1998). *I. obesulus* is primarily threatened by introduced predators, such as Red foxes (*Vulpes vulpes*, Linnaeus 1758) and feral cats (*Felis catus*, Linnaeus 1758) (Woinarski and Burbidge, 2014), and even sensitive to exotic non-predators (Randall et al., 2023), however they can persist in the presence of predators when appropriate habitat is provided. *I. obesulus* prefers woodland areas with a dense understory, particularly in the <1m stratum (Haby et al., 2013), although they will inhabit exotic, non-woodland areas provided sufficient density cover is available (Maclagan et al., 2020). *I. obesulus* is also sensitive to climate change (Rees et al., 2023), and climatic factors appear to have been the cause of ancestral and ongoing range contractions for the greater *Isoodon* genus (Paull et al., 2013; Pope et al., 2001), with *I. obesulus* showing a preference for high soil moisture (Paull, 2003), possibly implicating food availability as a limiting factor. These contractions have also been accelerated more recently with post-colonial habitat clearing (Paull et al., 2013). While increasing aridification is currently limiting *I. obesulus* distribution, if the climatic trends reduce aridity due to increased rainfall events or intensity (Burt et al., 2016; Martel et al., 2021), *I. obesulus* may in fact respond positively. However, most current populations are in decline (Woinarski and Burbidge, 2014).

Appendix S1 References

Burt, T., Boardman, J., Foster, I., Howden, N., 2016. More rain, less soil: long-term changes in rainfall intensity with climate change. Earth Surf. Process. Landf. 41, 563–566. https://doi.org/10.1002/esp.3868

Haby, N.A., Conran, J.G., Carthew, S.M., 2013. Microhabitat and vegetation structure preference: an example using southern brown bandicoots (Isoodon obesulus obesulus). J. Mammal. 94, 801–812. https://doi.org/10.1644/12-MAMM-A-220.1

Maclagan, S.J., Coates, T., Hradsky, B.A., Butryn, R., Ritchie, E.G., 2020. Life in linear habitats: the movement ecology of an endangered mammal in a peri-urban landscape. Anim. Conserv. 23, 260–272. https://doi.org/10.1111/acv.12533

Mallick, S.A., Driessen, M.M., Hocking, G.J., 1998. Biology of The Southern Brown Bandicoot (Isoodon Obesulus) In South-Eastern Tasmania. II. Demography. Aust. Mammal. 20, 339–347. https://doi.org/10.1071/am98339

Paull, D.J., 2003. Habitat fragmentation and the southern brown bandicoot Isoodon obesulus at multiple spatial scales. Doctoral thesis, University of New South Wales.

Paull, D.J., Mills, D.J., Claridge, A.W., 2013. Fragmentation of the Southern Brown Bandicoot Isoodon obesulus: Unraveling Past Climate Change from Vegetation Clearing. Int. J. Ecol. 2013, e536524. https://doi.org/10.1155/2013/536524

Pope, L., Storch, D., Adams, M., Moritz, C., Gordon, G., 2001. A phylogeny for the genus Isoodon and a range extension for I. obesulus peninsulae based on mtDNA control region and morphology. Aust. J. Zool. 49, 411–434. https://doi.org/10.1071/ZO00060

Randall, G.M., Weston, M.A., Rypalski, A., Rendall, A.R., 2023. Interactions between European rabbits and native marsupials in the absence of terrestrial predators. Austral Ecol. 0. https://doi.org/10.1111/aec.13281

Rees, M., Paull, D., 2000. Distribution of the southern brown bandicoot (Isoodon obesulus) in the Portland region of south-western Victoria. Wildl. Res. 27, 539. https://doi.org/10.1071/WR99045

Stoddart, D.M., Braithwaite, R.W., 1979. A Strategy for Utilization of Regenerating Heathland Habitat by the Brown Bandicoot (Isoodon obesulus; Marsupialia, Peramelidae). J. Anim. Ecol. 48, 165–179. https://doi.org/10.2307/4107

Woinarski, J.C.Z., Burbidge, A.A., 2014. IUCN Red List of Threatened Species: Isoodon obesulus. IUCN Red List Threat. Species.
