# Supplementary material for: Simulating Genetic Mixing in Strongly Structured Populations of the Threatened Southern Brown Bandicoot (Isoodon obesulus)
Source: Evol Appl. 2024 Dec 5;17(12):e70050. doi: 10.1111/eva.70050 (PMC11621039; doi:10.1111/eva.70050)
Supplement: Supplementary file 2 — Data S1. [file EVA-17-e70050-s001.docx]

# Supplementary Figures and Tables


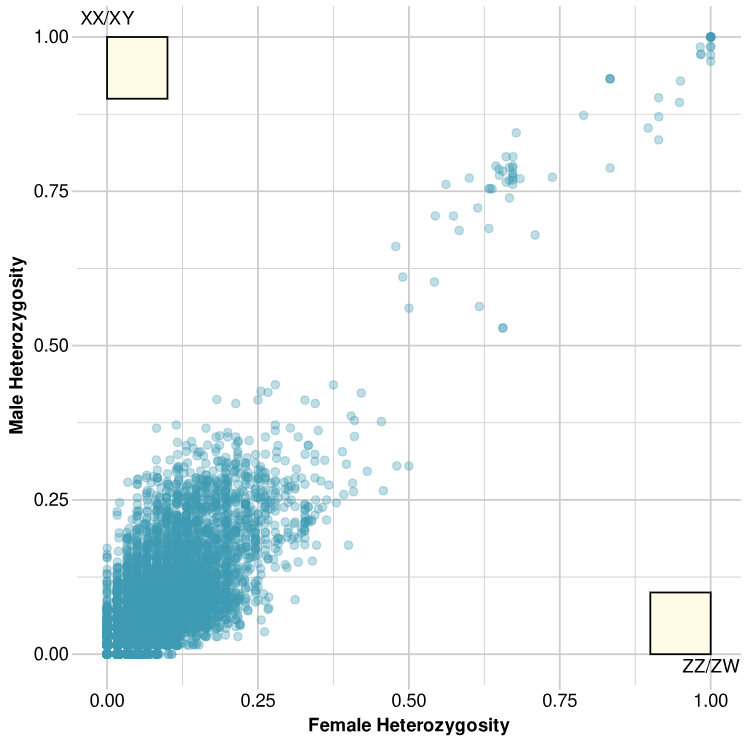


Suppl. Figure1: Scatterplot of mean female and male heterozygosity for each heterozygous locus in 132 *Isoodon obesulus* with sex metadata, produced by gl.report.sexlinked from the package *dartR*. Yellow squares indicate heterozygosity values typical of sex-linked loci.

Suppl. Table1: Assessment of Mn values for *de novo* alignment of 184 *Isoodon obesulus* samples.

| Mn parameter | Genotyped loci | Mean coverage | Min coverage | Max coverage | Mean sites per locus |
| --- | --- | --- | --- | --- | --- |
| 1 | 397700 | 31.1 | 6 | 44.5 | 80.1 |
| 2 | 365071 | 31.4 | 6 | 44.7 | 80.2 |
| 3 | 355210 | 31.6 | 6.1 | 45 | 80.2 |
| 4 | 349386 | 31.6 | 6.1 | 45 | 80.2 |
| 5 | 346025 | 31.6 | 6.2 | 45.1 | 80.2 |
| 6 | 343514 | 31.7 | 6.2 | 45.1 | 80.2 |


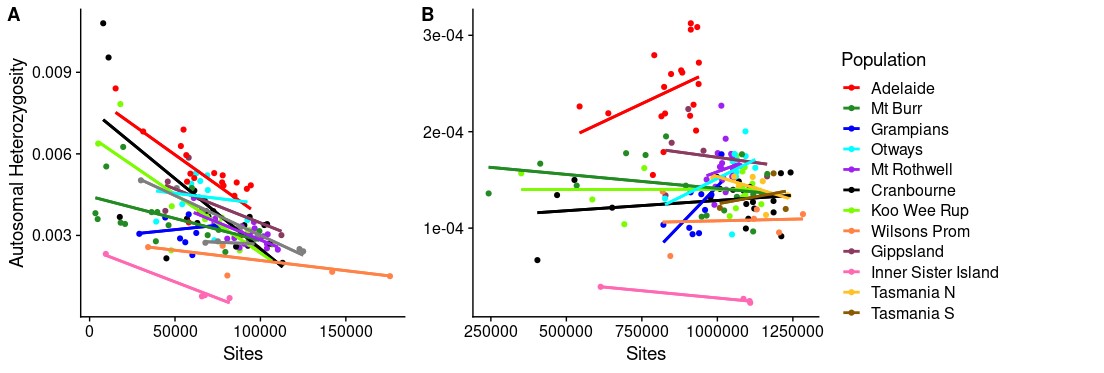


Suppl. Figure2: Individual autosomal heterozygosity and total retained sites in 137 *Isoodon obesulus* in south-eastern Australia. **A)** Shows per-population correlations between observed autosomal heterozygosity and observed sites for a pipeline with samples run as a single batch. 5 per-population correlations were found to be statistically significant after Bonferroni correction for multiple comparisons at α < 0.05 (p < 0.0041), and 1 observation at 0.05 < α < 0.1 (0.0041 < p < 0.0083). **B)** Shows per-population correlations between observed autosomal heterozygosity and observed sites for a pipeline with samples run individually with final filters. No correlations were found to be significant after Bonferroni correction for multiple comparisons at α < 0.05.


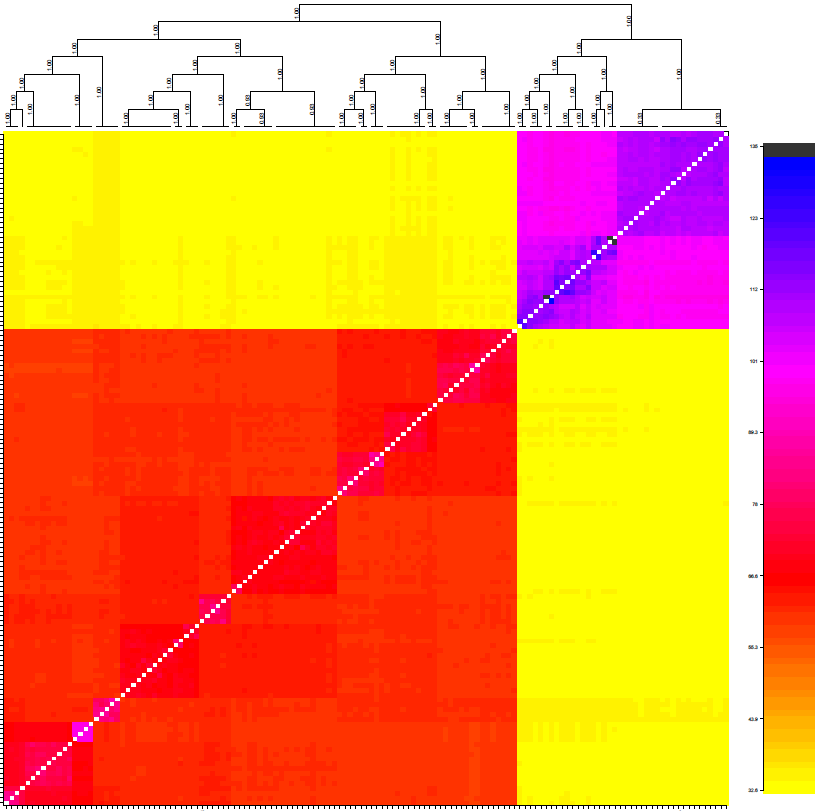

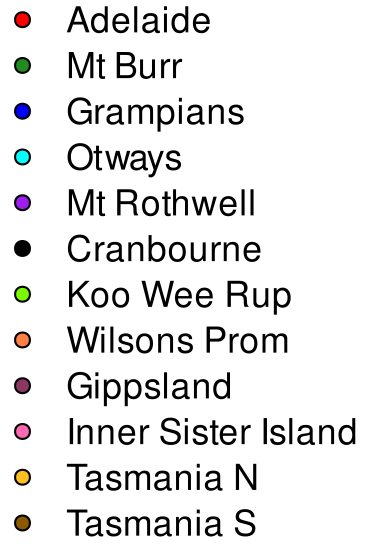

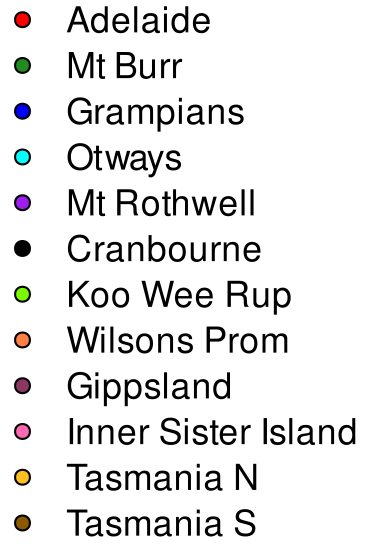

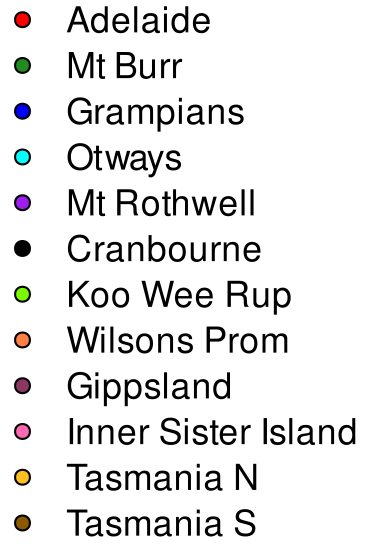

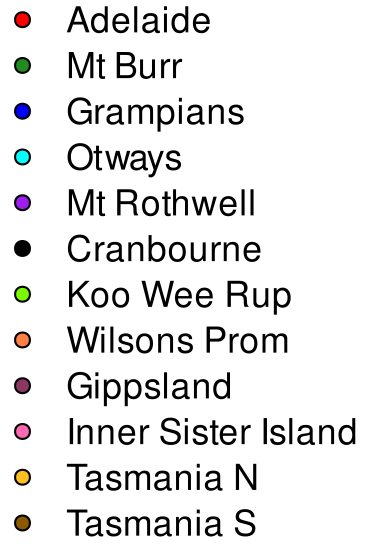

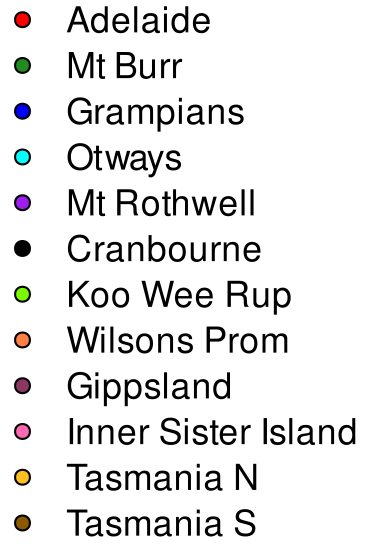

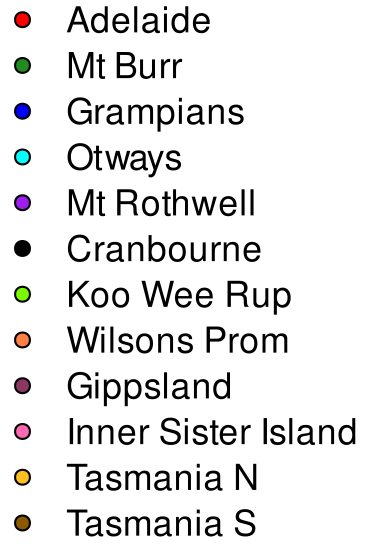

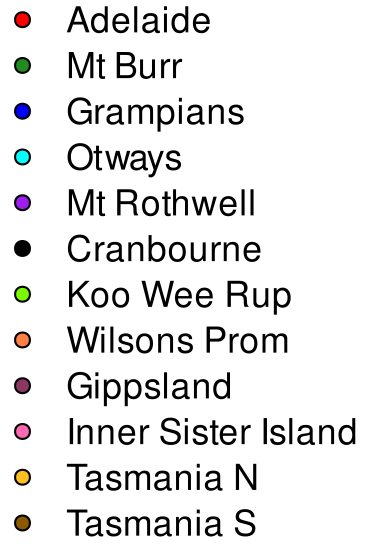

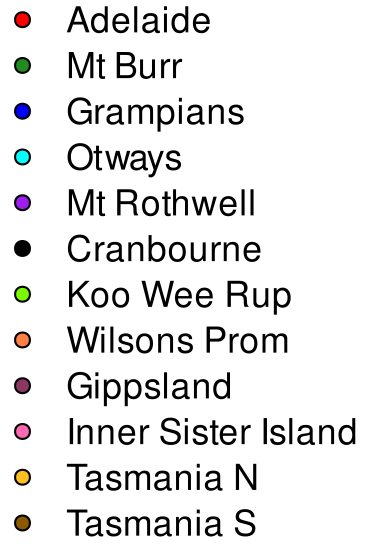

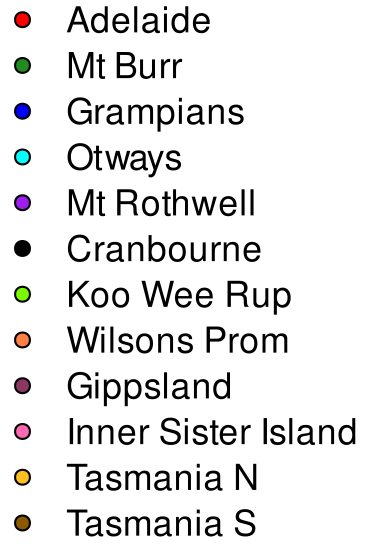

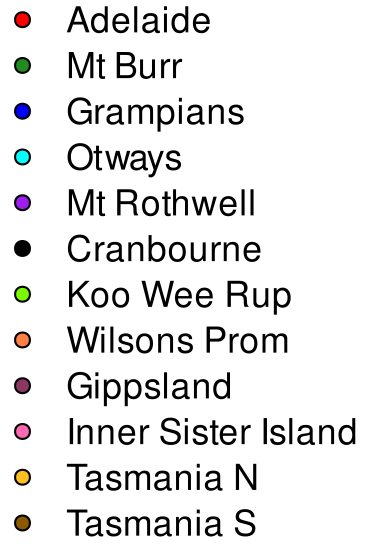

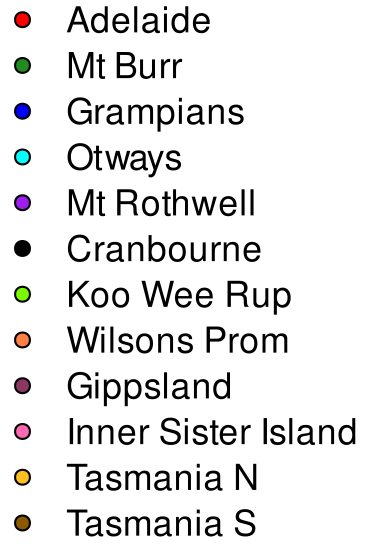

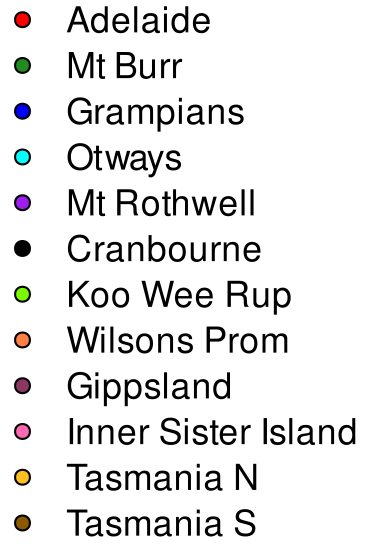


Suppl. Figure3: Heatmap of coancestry coefficients generated by *fineRADstructure* 137 *Isoodon obesulus*. Cells indicate an estimate of shared coancestry between two individuals and are shaded from yellow (low) to blue (high) to indicate increasing levels of coancestry. Above is a clustering dendrogram based on the matrix of coancestry coefficients, estimating population divergence points, with posterior probability on arms.


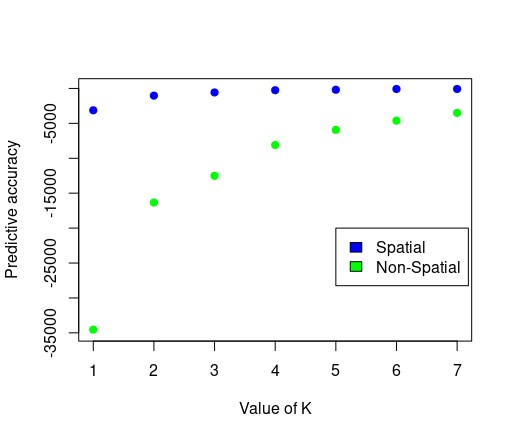
Suppl. Suppl. Figure4: *ConStruct* cross-validation test of various values of K in clustering models that account for spatial decay of genotype similarity (blue) and models that do not account for spatial decay (green). Predictive accuracy indicates deviation from the model of highest mean accuracy, which is scored as 0.


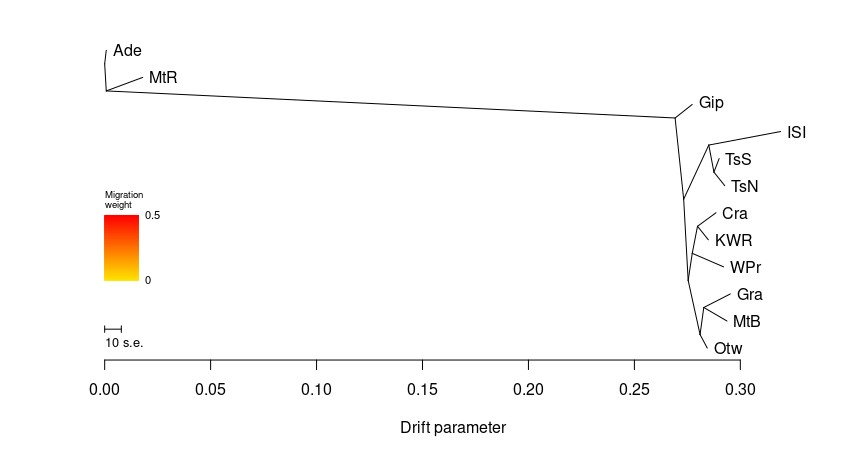


Suppl. Figure5: *TreeMix* dendrogram of relative drift between populations of 171 *Isoodon obesulus* across southeastern Australia, with Adelaide Hills assigned the root and zero migration fronts allowed.

Suppl. Table2: Tukey’s test for Honestly Significant Differences between individual autosomal heterozygosity in *Isoodon obesulus* populations in south-eastern Australia. Only statistically significant values at α’ = 0.05 after adjustment for multiple comparisons are shown.

| Population 1 | Population 2 | Difference in means  (Pop1 – Pop 2) | Upper 95% CI | Lower 95% CI | Adjusted P |
| --- | --- | --- | --- | --- | --- |
| Adelaide | Cranbourne | 0.00011 | 0.00014 | 0.00009 | 0.00000 |
| Adelaide | Gippsland | 0.00007 | 0.00011 | 0.00002 | 0.00019 |
| Adelaide | Grampians | 0.00012 | 0.00016 | 0.00008 | 0.00000 |
| Adelaide | Inner Sister Island | 0.00021 | 0.00026 | 0.00016 | 0.00000 |
| Adelaide | Koo Wee Rup | 0.00010 | 0.00013 | 0.00007 | 0.00000 |
| Adelaide | Mt Burr | 0.00010 | 0.00013 | 0.00007 | 0.00000 |
| Adelaide | Mt Rothwell | 0.00008 | 0.00011 | 0.00005 | 0.00000 |
| Adelaide | Otways | 0.00008 | 0.00012 | 0.00005 | 0.00000 |
| Adelaide | Tasmania N | 0.00010 | 0.00014 | 0.00006 | 0.00000 |
| Adelaide | Tasmania S | 0.00011 | 0.00016 | 0.00006 | 0.00000 |
| Adelaide | Wilsons Prom | 0.00014 | 0.00018 | 0.00009 | 0.00000 |
| Cranbourne | Gippsland | -0.00005 | -0.00000 | -0.00009 | 0.03180 |
| Cranbourne | Inner Sister Island | 0.00010 | 0.00015 | 0.00005 | 0.00000 |
| Cranbourne | Mt Rothwell | -0.00003 | 0.00000 | -0.00006 | 0.00826 |
| Gippsland | Grampians | 0.00005 | 0.00011 | 0.00000 | 0.02474 |
| Gippsland | Inner Sister Island | 0.00015 | 0.00021 | 0.00009 | 0.00000 |
| Gippsland | Wilsons Prom | 0.00007 | 0.00012 | 0.00001 | 0.00400 |
| Grampians | Inner Sister Island | 0.00009 | 0.00015 | 0.00004 | 0.00001 |
| Grampians | Mt Rothwell | -0.00004 | 0.00000 | -0.00008 | 0.01828 |
| Inner Sister Island | Koo Wee Rup | -0.00011 | -0.00006 | -0.00016 | 0.00000 |
| Inner Sister Island | Mt Burr | -0.00012 | -0.00007 | -0.00017 | 0.00000 |
| Inner Sister Island | Mt Rothwell | -0.00013 | -0.00008 | -0.00018 | 0.00000 |
| Inner Sister Island | Otways | -0.00013 | -0.00008 | -0.00018 | 0.00000 |
| Inner Sister Island | Tasmania N | -0.00011 | -0.00006 | -0.00017 | 0.00000 |
| Inner Sister Island | Tasmania S | -0.00011 | -0.00004 | -0.00017 | 0.00002 |
| Inner Sister Island | Wilsons Prom | -0.00008 | -0.00002 | -0.00014 | 0.00082 |
| Mt Rothwell | Wilsons Prom | 0.00005 | 0.00010 | 0.00001 | 0.00258 |
| Otways | Wilsons Prom | 0.00005 | 0.00010 | 0.00000 | 0.02127 |


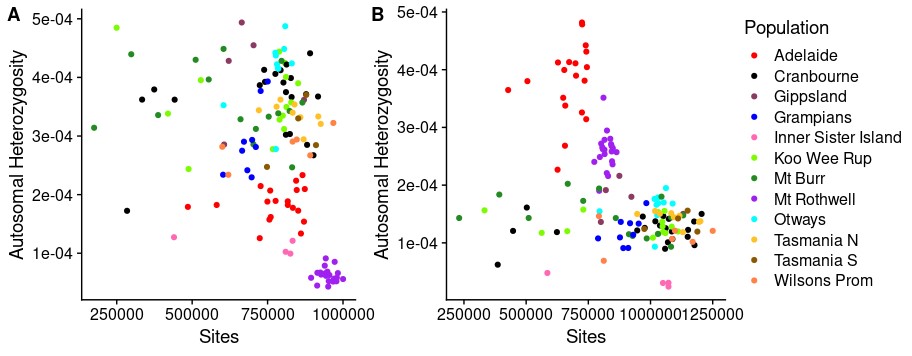


Suppl. Figure 6: Individualized autosomal heterozygosity predictions for *I. obesulus*, when the pseudo-reference used for variant calling was restricted to **A)** only samples of Greater Adelaide origin, and **B)** all samples outside of Greater Adeliade.
